# Vertebral Pneumaticity in the Ornithomimosaur *Archaeornithomimus* (Dinosauria: Theropoda) Revealed by Computed Tomography Imaging and Reappraisal of Axial Pneumaticity in Ornithomimosauria

**DOI:** 10.1371/journal.pone.0145168

**Published:** 2015-12-18

**Authors:** Akinobu Watanabe, Maria Eugenia Leone Gold, Stephen L. Brusatte, Roger B. J. Benson, Jonah Choiniere, Amy Davidson, Mark A. Norell

**Affiliations:** 1 Division of Paleontology, American Museum of Natural History, New York, New York, United States of America; 2 Richard Gilder Graduate School, American Museum of Natural History, New York, New York, United States of America; 3 School of GeoSciences, University of Edinburgh, Scotland, United Kingdom; 4 Department of Earth Sciences, University of Oxford, Oxford, United Kingdom; 5 Evolutionary Studies Institute and DST/NRF Centre of Excellence in Palaeosciences, University of the Witwatersrand, Johannesburg, South Africa; College of the Holy Cross, UNITED STATES

## Abstract

Among extant vertebrates, pneumatization of postcranial bones is unique to birds, with few known exceptions in other groups. Through reduction in bone mass, this feature is thought to benefit flight capacity in modern birds, but its prevalence in non-avian dinosaurs of variable sizes has generated competing hypotheses on the initial adaptive significance of postcranial pneumaticity. To better understand the evolutionary history of postcranial pneumaticity, studies have surveyed its distribution among non-avian dinosaurs. Nevertheless, the degree of pneumaticity in the basal coelurosaurian group Ornithomimosauria remains poorly known, despite their potential to greatly enhance our understanding of the early evolution of pneumatic bones along the lineage leading to birds. Historically, the identification of postcranial pneumaticity in non-avian dinosaurs has been based on examination of external morphology, and few studies thus far have focused on the internal architecture of pneumatic structures inside the bones. Here, we describe the vertebral pneumaticity of the ornithomimosaur *Archaeornithomimus* with the aid of X-ray computed tomography (CT) imaging. Complementary examination of external and internal osteology reveals (1) highly pneumatized cervical vertebrae with an elaborate configuration of interconnected chambers within the neural arch and the centrum; (2) anterior dorsal vertebrae with pneumatic chambers inside the neural arch; (3) apneumatic sacral vertebrae; and (4) a subset of proximal caudal vertebrae with limited pneumatic invasion into the neural arch. Comparisons with other theropod dinosaurs suggest that ornithomimosaurs primitively exhibited a plesiomorphic theropod condition for axial pneumaticity that was extended among later taxa, such as *Archaeornithomimus* and large bodied *Deinocheirus*. This finding corroborates the notion that evolutionary increases in vertebral pneumaticity occurred in parallel among independent lineages of bird-line archosaurs. Beyond providing a comprehensive view of vertebral pneumaticity in a non-avian coelurosaur, this study demonstrates the utility and need of CT imaging for further clarifying the early evolutionary history of postcranial pneumaticity.

## Introduction

Aves, a group comprising the last common ancestor of all extant birds and all of its descendants [1], exhibits a suite of specialized characteristics, including flight feathers, highly elongated forelimbs, and extensive skeletal fusion in the limbs and skull [2]. Another key feature observed in modern birds is postcranial pneumaticity, where respiratory passages (diverticula) derived from pulmonary air sacs invade bones such as vertebrae, ribs, and portions of the appendicular skeleton [3–5]. Postcranial pneumaticity is absent in all other extant vertebrates with the known exceptions of osteoglossomorph fish [6] and the hyoid bone in howler monkeys [7,8]. Although it does not contribute directly to pulmonary respiration [9], postcranial pneumaticity replaces metabolically costly bone, reducing bone mass and metabolic energy consumption, which may have enabled energetically demanding flight capabilities [10,11].

As with many other traits traditionally attributed to birds, the evolutionary origin of postcranial pneumaticity precedes the origin of birds, first appearing among pterosaurs [12–15] and non-avian dinosaurs (e.g., [2,16–18]). Because it appears in clearly non-volant, non-avian dinosaurs, selection for flight capacity certainly cannot explain the origins and early evolution of postcranial pneumaticity. Although large body size may have necessitated bone mass reduction in sauropods and non-maniraptoran theropods [17,19,20], relatively small non-avian maniraptorans also evolved substantial increases in the proportion of pneumatized postcranial bones [17]. As such, other factors have also been proposed to explain the early evolution of postcranial pneumaticity, including heightened metabolic needs [16,21,22], locomotory balance [23], and thermoregulation [19,22,24]. In addition, the distribution of postcranial pneumaticity has been used as an osteological correlate for the presence of the specific pulmonary air sacs that underlie the unique respiratory system in modern birds [16].

To elucidate the evolutionary origin of pneumatic structures, a comprehensive survey of postcranial pneumaticity in bird-line dinosaurs is critical. Benson and colleagues [17] documented the presence of vertebral pneumaticity across theropods, providing an extensive taxonomic sample. However, as with most studies of vertebral pneumaticity in non-avian dinosaurs, the identification of pneumaticity was entirely based on whether a foramen that connects with an internal chamber is visible on the bones without a full characterization of internal pneumatic structures. This approach resulted in substantial missing data for some clades, particularly for ornithomimosaurs. Here, we attempt to clarify enigmatic patterns of vertebral pneumaticity in ornithomimosaurs by applying micro-computed tomography (μCT) imaging to the basal, late Cretaceous ornithomimid *Archaeornithomimus* [25,26]. CT imaging enables reconstruction and visualization of internal pneumatic structures that cannot be observed externally (e.g., [14,15] for pterosaurs), but it has been seldom used to study postcranial pneumaticity in non-avian theropods (e.g., [12,27]).

*Archaeornithomimus* is known from an abundance of relatively intact, three-dimensionally preserved fossil specimens. As a major theropod clade, diverging close to the base of Coelurosauria, ornithomimosaurs constitute an important group for understanding the early evolution of features traditionally attributed to birds [2,18,28]. Ornithomimosaurs spanned three orders of magnitude in body size [29], from an estimated 5.3 kg (*Nqwebasaurus* [30]) to 620 kg (*Beishanlong* [26]) and exceptionally in excess of 6000 kg (*Deinocheirus* [31]). Phylogenetic ghost lineages imply that ornithomimosaurs had originated by the Middle Jurassic, and attained a broad distribution during the Cretaceous [32], including paleo-arctic [33] and Gondwanan [30] occurrences. In this study, we provide a comprehensive description of the vertebral pneumaticity of *Archaeornithomimus* and survey the degree of pneumaticity in other ornithomimosaurs to determine the macroevolutionary pattern of vertebral pneumaticity in Ornithomimosauria.

## Materials and Methods

### Specimens

Specimens examined in this study are from the Albany Museum, Grahamstown, South Africa (AM), American Museum of Natural History, New York, USA (AMNH), Las Hoyas Collection, Universidad Autónoma de Madrid, Madrid, Spain (LH), National Geological Museum of China, Beijing, People's Republic of China (NGMC), Royal Ontario Museum, Toronto, Canada (ROM), and the Institute of Paleobiology, Warsaw, Poland (ZPAL). These specimens are in permanent repository accessible to other researchers. The specimens sampled from respective institutions include AM 6040; AMNH 21786, 21788, 21790, 21794, 21802; LH 7777; NGMC 97-4-002; ZPAL MgD-I/1, MgD-I/7, MgD-I/8, MgD-I/39, MgD-I/94, MgD-I/207; ROM 851.

We selected representative and best-preserved vertebrae of *Archaeornithomimus asiaticus* Gilmore, 1933, including one cervical (AMNH FARB 21786), two dorsal (AMNH FARB 21788), two sacral (AMNH FARB 21790), four proximal caudal (AMNH FARB 21790, 21802), and three distal caudal vertebrae (AMNH FARB 21794). These specimens are from the Upper Cretaceous Iren Dabasu Formation of Inner Mongolia [34] and were discovered in 1923 by Peter Kaisen during the AMNH Third Central Asiatic Expedition led by Roy Chapman Andrews. The vertebrae are from multiple individuals excavated from different quarries but in close proximity. Smith and Galton [35] provided a brief description of the external morphology of these and other *Archaeornithomimus* elements. Here, we report additional morphological features revealed through CT imaging and further mechanical preparation of the vertebrae.

### Computed Tomography Imaging

The vertebrae were imaged with a GE phoenix v|tome|x micro-CT scanner at the AMNH Microscopy and Imaging Facility. Each vertebra, or articulated set of vertebrae, was scanned with the following parameters: voltage of 170–220 kV, current of 150–260 μA, and voxel size between 84.9 and 120.9 μm (S1 Table). Visual Graphics Studio Max version 2.2 (Volume Graphics GmbH, Heidelberg, Germany) was used to examine the internal structures depicted in the scan images and to construct three-dimensional digital renderings of the specimens.

### Mechanical Preparation

The original preparation was crudely done, with some damage from a grinder, and a heavy, yellowed coating had been applied over much of the specimen. Grey plaster had been used to join, fill and sculpt over much of the left side of the dorsal vertebrae (AMNH FARB 21788), particularly the centrodiapophyseal lamina ventral to the transverse processes. One of us (AD) re-prepared areas of interest by removing the coating, matrix, and plaster overlaying intact bone, using needles, airscribes and a minigrinder. The right prezygopophysis of the anterior dorsal vertebra, visible in the CT scan, was determined to be a fragment suspended in matrix with no bony contact. It was removed in order to gain access to the underlying fossa.

### Survey within Ornithomimosauria

In attempt to infer macroevolutionary trends in vertebral pneumaticity within Ornithomimosauria, we updated the characterization of pneumaticity in ornithomimosaurs based on literature and personal observations (all specimens examined are in permanent collections at respective institutions). For the optimization of the evolution of axial pneumaticity, we used a composite phylogeny based on several previous works [26,28,30,31,36]. This includes a basal grade comprising *Nqwebasaurus*, *Pelecanimimus*, and *Shenzhousaurus* [30] as successively proximate outgroups to a clade comprising Deinocheridae (after [31]) and the widely accepted Ornithomimidae. Deinocheiridae consists of *Beishanlong*, *Garudimimus*, and the giant *Deinocheirus* [31]. Ornithomimidae comprises *Ornithomimus*, *Struthiomimus*, *Gallimimus*, *Anserimimus* and *Dromiceiomimus*, supported by consensus among previous studies. The phylogenetic positions of *Sinornithomimus* and *Harpymimus* are unstable, and thus, these taxa were removed from analysis. *Archaeornithomimus* shares the derived, arctometatarsalian condition with ornithomimids [25,35]. In phylogenetic analyses, Kobayashi and Lü [36] found it as an early diverging ornithomimid, and Makovicky and colleagues [26] showed it to be in a polytomy with the other ornithomimids. We follow the conclusion of Kobayashi and Lü [36] by placing *Archaeornithomimus* within Ornithomimidae.

### Terminology

In this study we follow the criteria recommended by O’Connor [24] and consider the joint presence of (1) large internal chambers and (2) a pneumatic foramen linking the chambers to the external surface of the bone as unambiguous evidence for pneumaticity. More ambiguous osteological correlates include internal chambers where clear external communication cannot be confirmed, in some cases due to taphonomic damage. Anatomical nomenclature of vertebral laminae and fossae follow Wilson [37,38], and we employ terminology proposed by Wedel and colleagues [39] to summarize pneumatic structures present on the vertebrae. We use the definitions provided by Britt [24] to describe internal pneumatic structures. Specifically, ‘camerate’ refers to a system of large internal chambers divided by major septa within bones with thick external walls and ‘camellate’ refers to a system of small internal chambers within vertebrae with thin external walls. These conditions are explicitly end-members of a continuous and potentially quantifiable spectrum, with various saurischian taxa showing a range of intermediate conditions (e.g., [37]).

## Description

### Cervical vertebra

**Fig 1:**
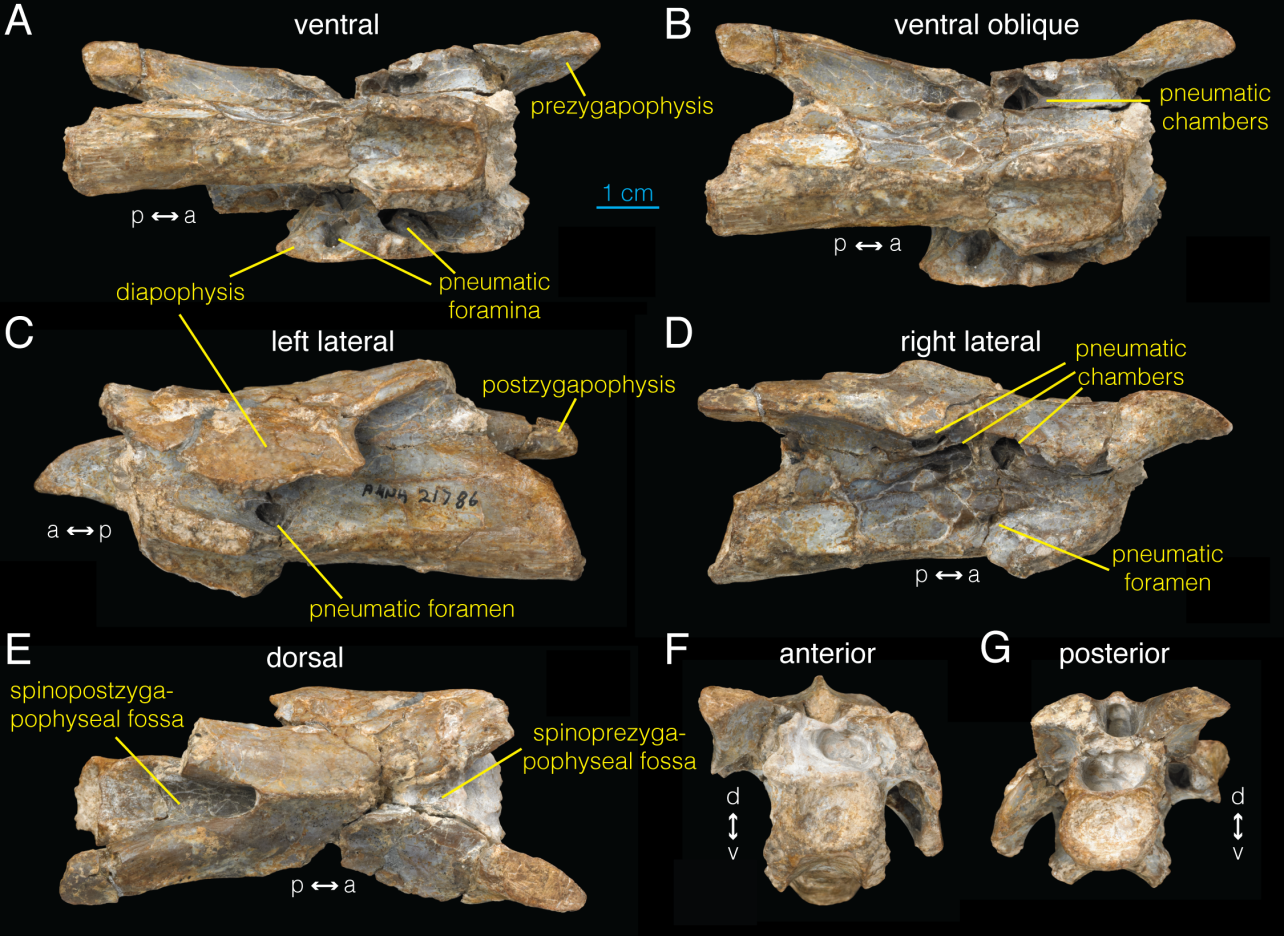
Postaxial cervical vertebra of *Archaeornithomimus* (AMNH FARB 21786). **A**, ventral; **B**, ventral oblique view; **C**, left lateral; **D**, right lateral; **E**, dorsal; **F**, anterior; **G**, posterior view.

Further mechanical preparation of the mid-cervical vertebra (AMNH FARB 21786), tentatively assigned to cervical 5 [33], reveals several foramina and interconnected pneumatic chambers on the ventral surfaces of the left and right transverse processes. The left transverse process is largely intact and bears two ellipsoid foramina on the ventral surface of its base (Fig 1A). The posteriormost foramen extends into an internal chamber within the transverse process (Fig 1A). A very thin lamina, which is translucent under direct light, separates this chamber from the external surface of the bone. Although we cannot discern from external observation whether this internal chamber connects to other internal chambers, it is clear that the more anterior foramen passes medially into a large internal cavity.

The right transverse process is missing, but the broken area at its base exposes the internal structures in this region. Four primary chambers are present along the ventral margin of the diapophysis (Fig 1B), which were likely connected to equivalent foramina observed on the left transverse process. The chamber extending from the most posterior foramen only slightly invades the vertebra, without any connections to other internal chambers. However, the most anterior chamber extends anteriorly into three secondary chambers. The middle two chambers are circular in cross section, and give rise to an intricate network of internal chambers divided by thin laminae.

In addition to the neural arch foramina, a pneumatic foramen (‘pleurocoel’) is present on the lateral surface of the centrum, located posterodorsal to the parapophysis and ventral to the preserved portion of the transverse process (anteroposterior diameter: 7.5 mm; Fig 1C). This foramen is housed within a fossa and leads into an internal chamber. Besides the centrum and transverse processes, an extensive spinopostzygapophyseal fossa is present directly dorsal to the neural canal on the posterior surface of the bone (Fig 1E and 1G).

**Fig 2:**
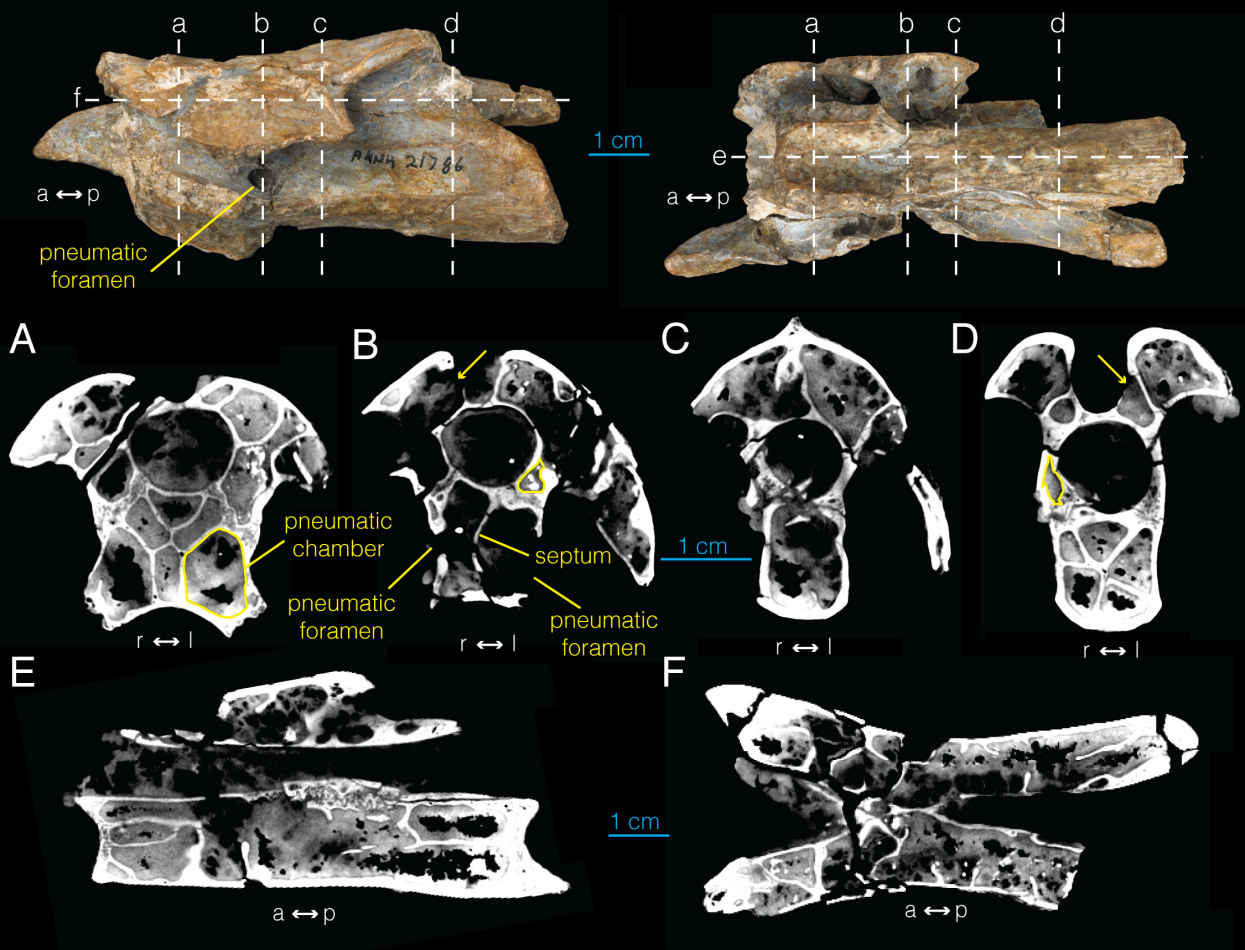
CT images of postaxial cervical vertebra of *Archaeornithomimus* (AMNH FARB 21786). **A–D**, select transverse sections; **E**, midsagittal section; **F**, frontal section. Dashed lines and associated letters indicate location and letter designation of CT image slices.

CT images of the cervical vertebra show an extensive pneumatic network inside the neural arch, transverse processes, and the centrum (Fig 2; S1 File). This network is mostly bilaterally symmetric and not visible externally with the exclusion of the chamber associated with the left pleurocoel (Fig 1C). In the anterior sections of the vertebra (Fig 2A), at least 13 distinct pneumatic chambers are visible in transverse view. This “camellate” condition comprises regularly branching, relatively large internal chambers [12,39,40]. Six distinct chambers are present that do not communicate with each other anterior to the pleurocoel. The two largest chambers are located laterally and ventrally within the centrum, have irregular, hexagonal cross sections, and extend dorsally approximately to the level of the neurocentral suture. Between these two chambers, a lower, narrower chamber is present which has a tall, ovoid cross section. Two chambers with rectangular cross section lie above the two ventrolateral chambers with another chamber with hemispherical cross section between them. These six chambers, along with additional irregularly distributed chambers, merge sporadically in the posterior direction within left and right sides, forming two larger chambers that occupy the centrum separated by a median septum at the level of the pleurocoel (Fig 2B).

The pneumatic foramen on the left side of the centrum leads into a single internal chamber that occupies the left ventrolateral section of the anterior end of the centrum, and is connected to the opposing chamber on the right side of the centrum via a foramen in the median internal septum (Fig 2B). A smaller pneumatic foramen of equivalent position on the right side of the centrum (Fig 1D) extends directly into the right ventrolateral chamber in the anterior centrum (Fig 2B). At the posterior margin of the left pneumatic foramen, these two chambers join to form a single pneumatic chamber that extends posteriorly to the level of the base of postzygapophysis (Fig 2C). More posteriorly, this chamber differentiates into asymmetric, pentaradial chambers (Fig 2D).

The internal structure of anterior neural arch is characterized by four principal chambers located dorsolateral and ventrolateral to the neural canal (Fig 2A). As the dorsoventral pair of chambers becomes obliterated at the level of pleurocoels, the ventrolateral pair expands dorsally to occupy this chamber (Fig 2B). More posteriorly, there is a brief interval of increased compartmentalization, followed by the presence of two large pneumatic chambers dorsolateral to the neural canal until the posterior margin of the neural spine (Fig 2C). In the postzygapophysis, these chambers become subdivided into two (Fig 2D), then three compartments more posteriorly. Frontal CT sections of the vertebra (Fig 2F; Video B in S1 File) demonstrate that many of the compartments inside the neural arch are interconnected. Although the pneumatic chambers inside the left transverse process are difficult to characterize due to fractures and damage, several interconnected chambers are visible.

The spinozygapophyseal fossa forms a tubular structure ventral to the neural spine, which connects with the left chamber in the neural arch (arrow in Fig 2D). More anteriorly, there is a potential foramen in the spinoprezygapophyseal fossa into the right chamber although its origin could be taphonomic (Fig 2B). A direct pneumatic connection is absent between the spinoprezygapophyseal and spinopostzygapophyseal fossae within the neural spine. Although generally bilaterally symmetric, several smaller pneumatic chambers are observed throughout the bone that exist only on one side (e.g., outlined in Fig 2B and 2D). The CT images reveal that the chambers inside the neural arch and centrum are distinct from each other.

Similar to the condition seen in *Allosaurus* [40], the structure of pneumatization in this cervical vertebra of *Archaeornithomimus* is intermediate between end member conditions occurring in vertebrae of a range of other taxa including carcharodontosaurids, tyrannosauroids, and oviraptorosaurs that exhibit dense networks of small internal chambers [12,16,27,40].

### Dorsal vertebrae

**Fig 3:**
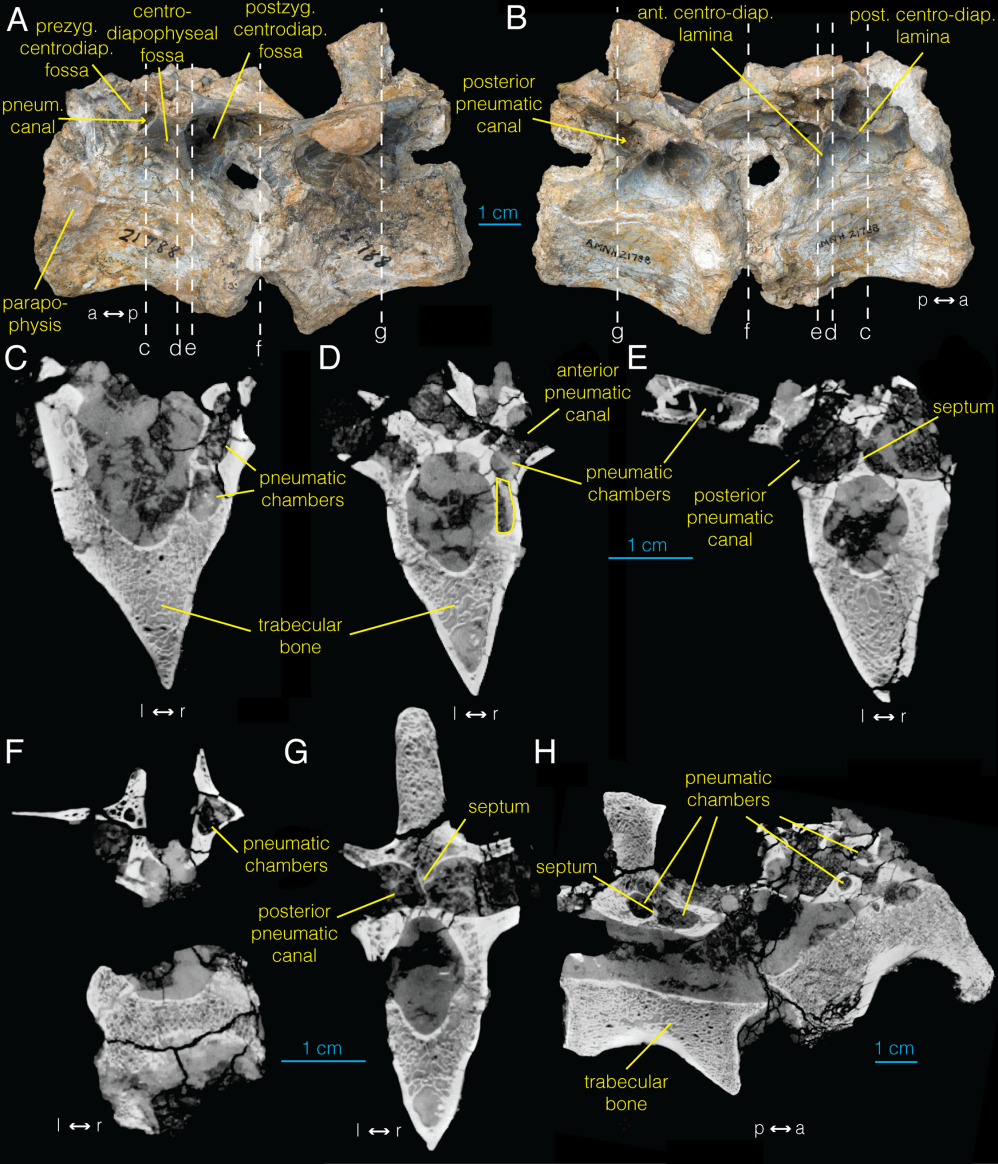
Anterior dorsal vertebrae of *Archaeornithomimus* (AMNH FARB 21788) and associated CT images. **A**, left lateral view; **B**, right lateral view; **C–G**, select transverse sections; **H**, midsagittal section. Dashed lines and associated letters indicate location and letter designation of CT image slices.

We examined two articulated dorsal vertebrae (AMNH FARB 21788; Fig 3), which Smith and Galton [35] designated as the two anteriormost dorsal vertebrae, without comment. In the more anterior vertebra, we observe a parapophysis that occupies both the centrum and neural arch (Fig 3A), denoting its position as the first dorsal vertebra. Unlike the cervical vertebra of *Archaeornithomimus*, these anterior dorsal vertebrae lack pneumatic features of the centrum such as large lateral foramina and internal pneumatic chambers. Internally, the centra of both vertebrae consist of trabecular bone with no sign of pneumaticity (Fig 3C–3E, 3G and 3H), unlike the pneumatized centrum of the cervical vertebra (Fig 2E). However, CT scans reveal that internal chambers are present in the neural arches (Fig 3D, 3E, 3G and 3H), and additional preparation uncovered several external features of the neural arches which communicate with these internal chambers and are therefore indicative of pneumatization according to the criteria of O’Connor [24].

A triangular centrodiapophyseal fossa lies immediately ventral to the transverse processes in both dorsal vertebrae. It is bound by anterior and posterior centrodiapophyseal laminae that buttress the transverse processes on both sides of the vertebra and separate the centrodiapophyseal fossa from prezygapophyseal and postzygapophyseal centrodiapophyseal fossae, respectively (Fig 3A and 3B). The dorsal area of the centrodiapophyseal fossa narrows medially, but this narrowing does not directly lead into internal pneumatic chambers. Instead, four very small foramina (diameter = 0.2–0.3 mm) are present within two of the centrodiapophyseal fossae. Two of these foramina are located ventrally within the narrowed portion of the left centrodiapophyseal fossa of the more anterior vertebra (S1 Fig) and two are located ventrally within right centrodiapophyseal fossa of the more posterior dorsal vertebra. These foramina do not appear to connect to internal pneumatic chambers in CT images, thus are designated to be neurovascular in origin. Additionally, a relatively large, foramen occurs in the posteroventral corner of this fossa, opening out posteriorly from the bone surface. The right postzygapophyseal centrodiapophyseal fossa also contains a dorsally opening ovoid foramen on its posterodorsal surface.

The neural arch exhibits a “procamerate” pattern [39], where both the prezygapophyseal and postzygapophyseal centrodiapophyseal fossae invade deeper to the median septum, forming funnel-shaped foramina (Fig 3A and 3B). In the more anterior vertebra of AMNH FARB 21788, these deep fossae meet medially to form an internal chamber (Fig 3D, 3E and 3G), which is not enclosed by bone compared to the camerae observed in the cervical vertebra. A thin median septum delineates the left and the right internal chambers, and is perforated by a small foramen located dorsally, connecting the left and right chambers (Fig 3E). However, whether this gap is biological or taphonomic is difficult to discern because of its relatively poor preservation. The more posterior dorsal vertebra of AMNH FARB 21788 also has a thin median lamina between the left and right chambers (Fig 3G) with three microscopic foramina perforating the septum (not figured), thereby connecting chambers across the midline although they may not have been linked pneumatically. In contrast to those in the more anterior vertebra however, the anterior and posterior chambers extending from the prezygapophyseal and postzygapophyseal centrodiapophyseal fossae respectively on either side are separated by a transversely oriented lamina (Fig 3H). Nevertheless, a small foramen is present in the lamina between the right prezygapophyseal and postzygapophyseal centrodiapophyseal fossae, suggesting that they may have been coupled pneumatically.

Beyond these easily visible pneumatic connections, additional smaller internal pneumatic canals further invade the dorsal vertebrae. In the more anterior vertebra, the right anterior canal within the prezygapophyseal centrodiapophyseal fossa contains an oval foramen that leads into a more anterior pneumatic chamber. The ventral floor of the left, as well as the intact surface of the right prezygapophyseal centrodiapophyseal fossae in the more posterior vertebra exhibit an undulating texture with widely distributed pits observed under light microscopy. In the more posterior vertebra, a deep sulcus extends from the posterior portion of this fossa. This sulcus extends dorsally, then anteriorly into a large and deep, matrix-filled fossa on the ventral surface of the transverse process between the prezygapophyseal centrodiapophyseal fossa and anterior centrodiapophyseal laminae. This pocket is inaccessible to mechanical preparation and CT images indicate that it does not lead into any other chambers inside the transverse process.

The CT data of AMNH FARB 21788 (Fig 3C–3H; S2 File) show that the more anterior vertebra exhibits an anteroposteriorly-elongated chamber within the peduncle of the right neural arch (Fig 3C and 3D). However, an equivalent chamber is absent on the left side. Anteriorly, the chamber bifurcates at the midpoint of the base of the prezygapophysis into dorsal and ventral chambers separated by a thin, horizontal bony strut (Fig 3C). The dorsal chamber continues from this bifurcation to the anterior-most point of the base of prezygapophysis. Similarly, the ventral chamber approaches the anterior margin of the base of prezygapophysis. Posteriorly, the ventral chamber extends to the longitudinal midpoint of the vertebra where it joins a larger cavity at the base of the transverse process (Fig 3D). The foramen that links to this internal pneumatic chamber lies ventral to the base of the transverse process in the prezygapophyseal and postzygapophyseal centrodiapophyseal fossae as described above.

A dorsoventrally compressed chamber is present inside the left transverse process (Fig 3E). Unfortunately, this transverse process is too damaged to discern a pneumatic foramen associated with this chamber. The right postzygapophysis also contains an anteroposteriorly elongate, internal chamber ellipsoid in transverse cross section (Fig 3F). Its anterior border is difficult to discern, but definitely extends laterally from the base of the postzygapophysis to the midpoint of the postzygapophysis. Anteriorly, this chamber is connected to the exterior of the bone via the postzygapophyseal centrodiapophyseal fossa (Fig 3E).

Unfortunately, the second, more posterior vertebra is too heavily damaged to identify additional evidence of pneumaticity in the CT images beyond the structures visible externally. Nevertheless, the transverse process of the posterior dorsal vertebra shows potential evidence of pneumaticity in the form of dorsoventrally compressed internal chamber (Fig 3G). This chamber, however, constitutes only ambiguous evidence of pneumaticity because a foramen linking the chamber to the external surface is absent. As in the centrum of the more anterior dorsal vertebra, the centrum of this vertebra is also almost entirely trabecular in composition (Fig 3G and 3H).

### Sacral vertebra

**Fig 4:**
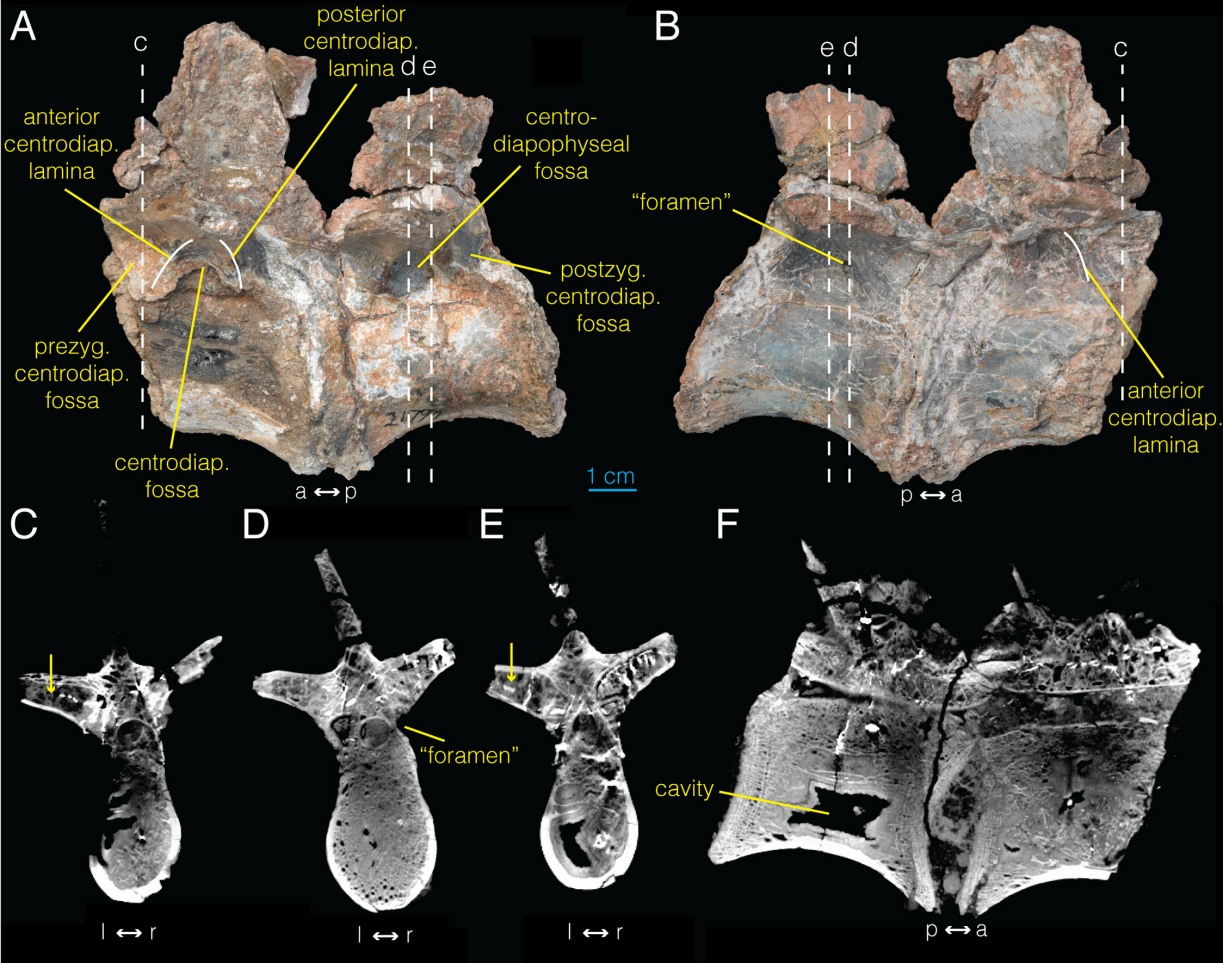
Sacral vertebrae of *Archaeornithomimus* (AMNH FARB 21790) and associated CT images. **A**, left lateral view; **B**, right lateral view; **C–E**, select transverse sections; **F**, midsagittal section. Dashed lines and associated letters indicate location and letter designation of CT image slices.

Based on manual articulation of disarticulated sacral vertebrae, we identify the two sacral vertebrae (AMNH FARB 21790) sampled for this study as the second and third sacral vertebrae. As in the dorsal vertebrae, the sacral vertebrae have centrodiapophyseal, as well as prezygapophyseal and postzygapophyseal centrodiapophyseal fossae (Fig 4A and 4B). On their own, the presence of these fossae constitutes only equivocal evidence of pneumaticity [21]. Pneumatic foramina are not visible on the surfaces of the more anterior sacral vertebrae. However, there is an ovoid foramen located in the left centrodiapophyseal fossa of the more posterior sacral vertebra (Fig 4B). This foramen, however, does not lead into any extensive internal chambers, and therefore is not clearly pneumatic in nature, being more likely a neurovascular foramen.

The CT imaging of the sacral vertebrae does not show any definitive signs of pneumaticity (Fig 4C–4F; S3 File). Much of the internal structure of the centra consists of trabeculae (Fig 4C). Whereas the right transverse process is almost entirely trabecular, the left transverse process of the more anterior sacral vertebra may contain an enlarged pneumatic chamber in its posterior half (arrow in Fig 4C). However, this is equivocal because the chamber is not clearly partitioned and it is not associated with a pneumatic foramen. Likewise, the transverse processes of the second, more posterior sacral vertebra exhibits a highly trabecular composition anteriorly and possible pneumaticity posteriorly based on the presence of an internal chamber (arrow in Fig 4E), but is not associated with the foramen observed on the external surface ventral to the right transverse process (Fig 4D). Similarly, the cavity observed in the centrum of the more posterior sacral vertebra (Fig 4F) is not associated with any pneumatic foramen. In summary, neither external morphology nor CT imaging of internal morphology provide any unambiguous evidence of pneumaticity in the sacral vertebrae of *Archaeornithomimus*. We, therefore, consider sacral vertebrae as “acamerate” [39], where fossae ventral to the transverse processes do not invade the vertebrae.

### Proximal caudal vertebrae

**Fig 5:**
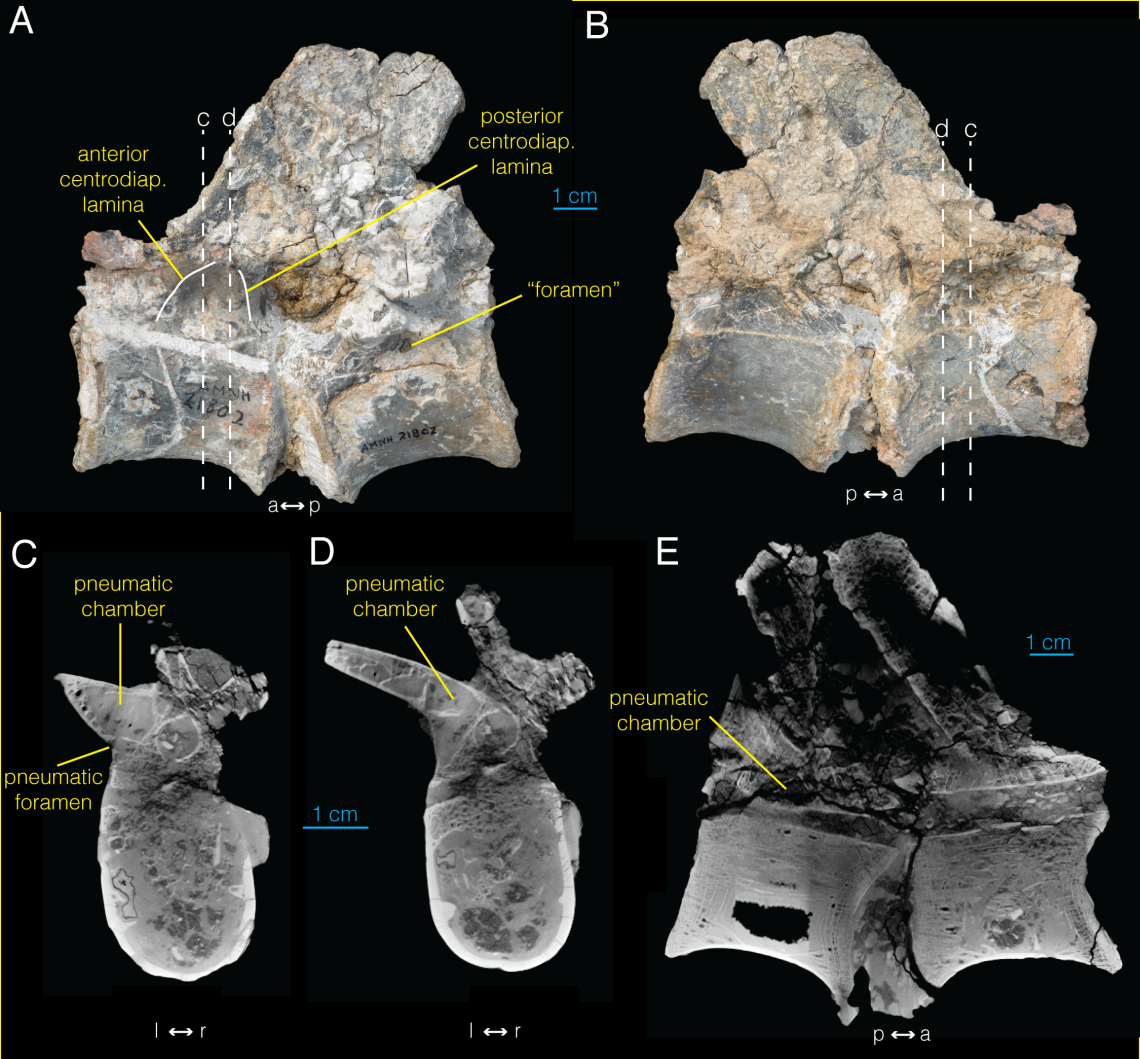
Proximal caudal vertebrae of *Archaeornithomimus* (AMNH FARB 21802) and associated CT images. **A**, left lateral view; **B**, right lateral view; **C, D**, select transverse sections; **E**, midsagittal section. Dashed lines and associated letters indicate location and letter designation of CT image slices.

**Fig 6:**
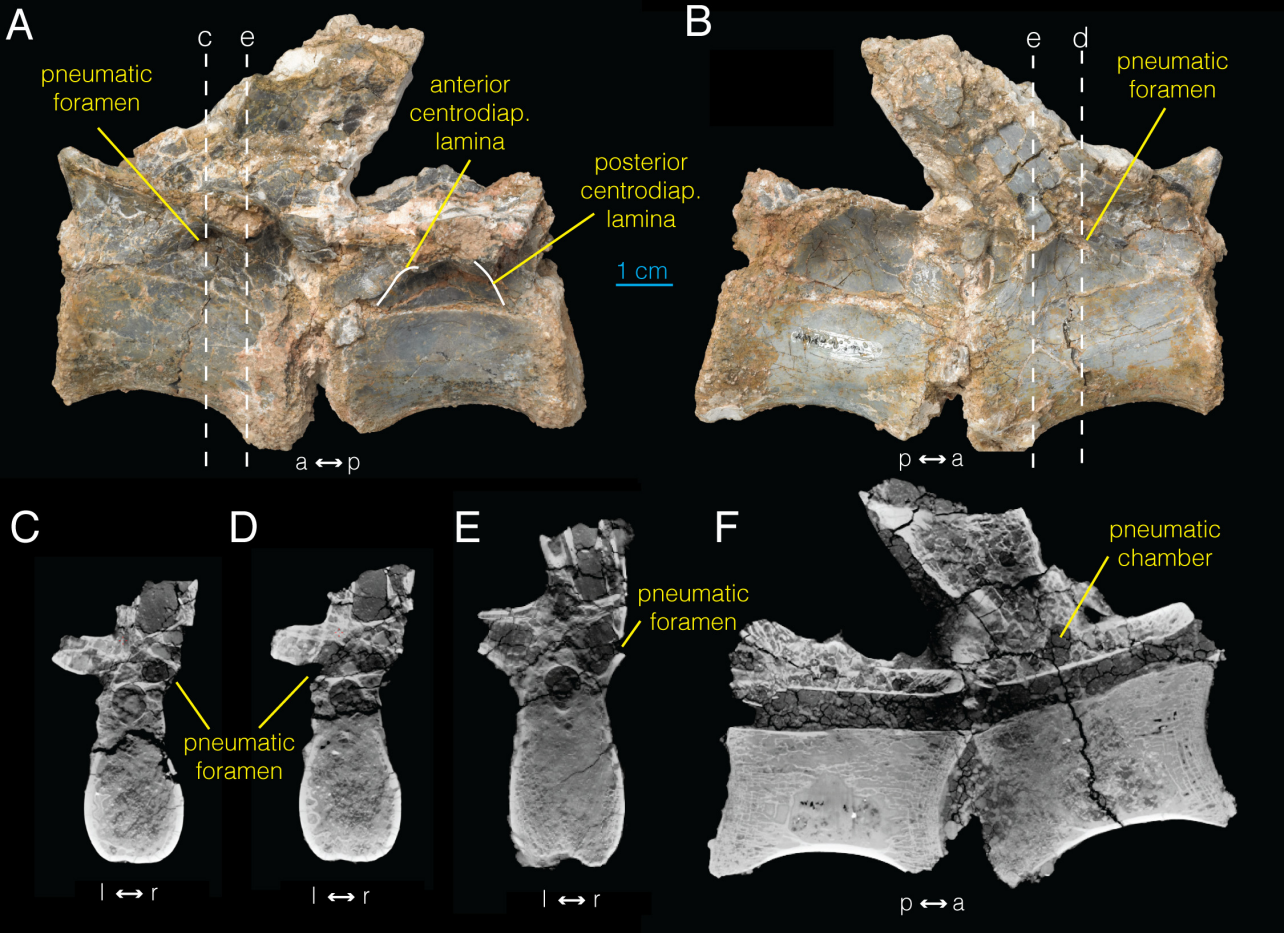
Proximal caudal vertebrae of *Archaeornithomimus* (AMNH FARB 21790) and associated CT images. **A**, left lateral view; **B**, right lateral view; **C–E**, select transverse section; **F**, midsagittal section. Dashed lines and associated letters indicate location and letter designation of CT image slices.

All four proximal caudal vertebrae have weakly depressed prezygapophyseal and postzygapophyseal centrodiapophyseal fossae (Figs 5A, 5B, 6A and 6B). The more posterior caudal vertebra in AMNH FARB 21802 displays a potential foramen ventral to the transverse process (Fig 5A). However, based on the presence of fractures in the surrounding area, this could easily have resulted from taphonomic damage, therefore not representing original morphology. Based on CT data, this opening is not associated with an internal chamber. Likewise, the left and right centrodiapophyseal fossae in the more anterior caudal vertebra of AMNH FARB 21790 exhibit foramina ventral to the transverse process on both sides (Fig 6A and 6B). The left foramen is more clearly defined and leads into at least one internal chamber at the base of the left transverse process (Fig 6C). Accordingly, we identify this opening as a true pneumatic foramen.

In CT data (S4 File), extensive chambers are present within both the neural arches and transverse processes of the more anterior caudal vertebra of AMNH FARB 21802 (Fig 5C and 5D) and the more posterior vertebra of AMNH FARB 21790 (Fig 6C–6F). In the more anterior vertebra of AMNH FARB 21802, large internal chambers are present at the base of the transverse processes on both sides (Fig 5C and 5D). Whether these chambers extend distally through the transverse process could not be determined as the area is damaged and only the bases of the processes remain. CT imaging reveals that a narrow opening is present on the lateral margin of the left cavity (Fig 5C). Upon closer inspection of the external surface, we observe two very small foramina (diameter <1 mm) in the preserved sections of the left transverse process that are associated with this chamber at its base.

Although the right transverse process is substantially damaged, the anterior vertebra of AMNH FARB 21790 shows a paired cavity inside the neural arch (Fig 6C–6F). This compartmentalized chamber occupies nearly the entire internal space of the neural arch from the base of the prezygapophyses to the base of the transverse processes. More posteriorly, trabecular bone obliterates the cavities (Fig 6F). Notably, both the left and right foramina observed in the centrodiapophyseal fossa of this vertebra connect to internal chambers (Fig 6C–6E). The left pneumatic foramen links directly to a relatively large internal chamber within the right neural arch (Fig 6D). Conversely, the right pneumatic foramen first connects into a small internal chamber immediately lateral to the neural canal, but joins a relatively large chamber dorsal and anterior to it (Fig 6C). The neural spine may is potentially pneumatic, but this is equivocal due to extensive damage in this region.

In all four proximal caudal vertebrae, the centrum is primarily trabecular internally (Fig 6F), albeit less vascularized than in dorsal and sacral vertebrae, and they lack any pneumatic chambers. The cavity in the centrum of the more posterior vertebra of AMNH FARB 21802 (Fig 5E) is not connected externally via a pneumatic foramen.

### Distal caudal vertebrae

**Fig 7:**
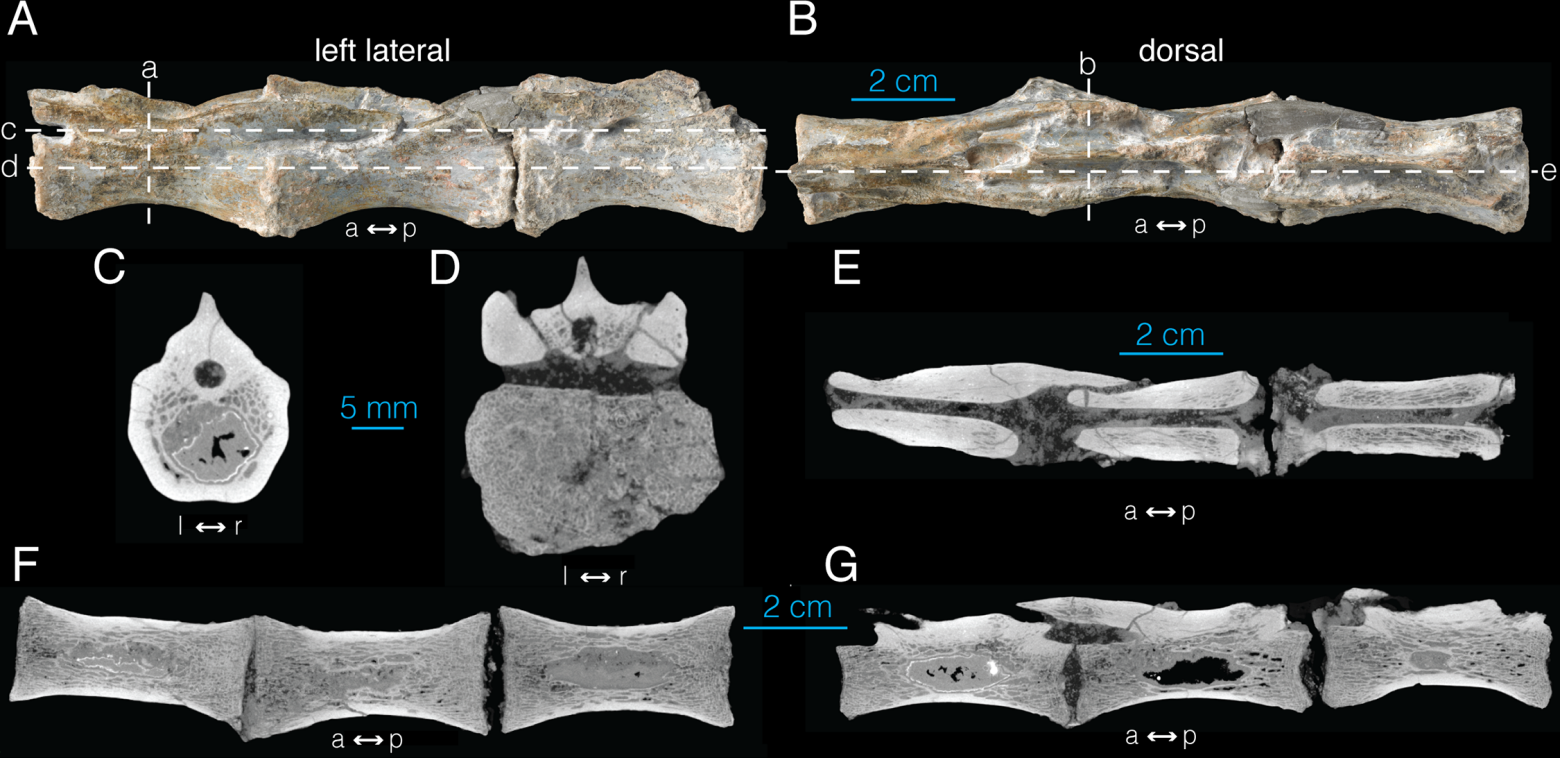
Distal caudal vertebrae of *Archaeornithomimus* (AMNH FARB 21794) and associated CT images. **A**, left lateral view; **B**, dorsal view; **C, D**, select transverse sections; **E**, frontal plane section; **F, G**, select sagittal sections. Dashed lines and associated letters indicate location and letter designation of CT image slices.

Despite the lack of potential pneumatic foramina on their external surface, three articulated distal caudal vertebrae (AMNH FARB 21794) were CT imaged. Due to incomplete series, the exact position of the cervical vertebrae is unknown. As expected, the CT data reveal complete absence of pneumaticity in the sampled caudal vertebrae (Fig 7).

## Survey of Ornithomimosaurs

### Basal ornithomimosaurs

#### Nqwebasaurus

**Fig 8:**
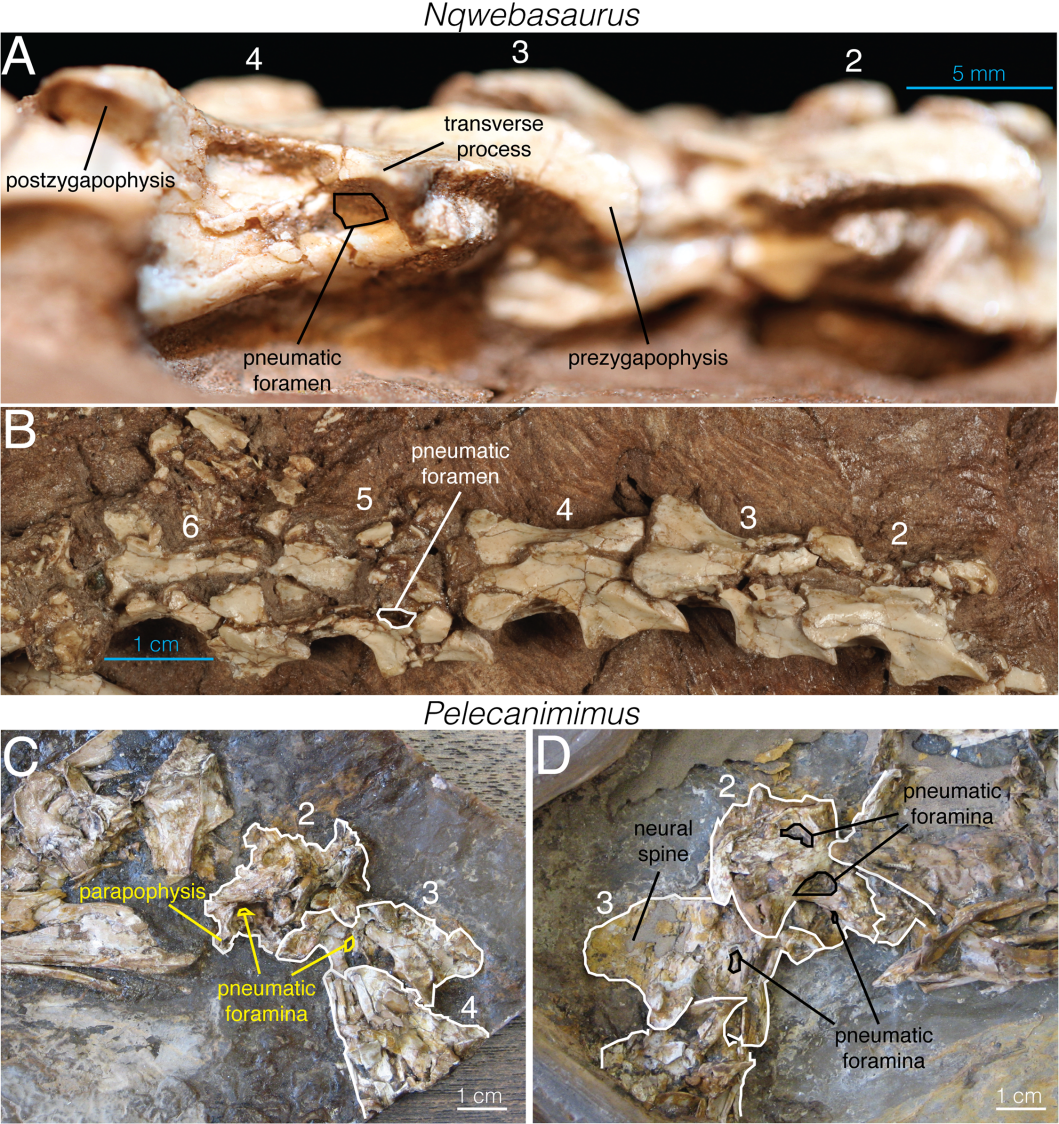
Cervical vertebrae of *Nqwebasaurus* (AM 6040) and *Pelecanimimus* (LH 7777). **A**, right lateral view; **B**, dorsal view of *Nqwebasaurus*. **C**, left lateral view; and **D**, right lateral view of *Pelecanimimus*. Numbers denote cervical vertebral number.

Nqwebasaurus is currently regarded as the oldest, earliest-branching, and skeletally the most completely known Gondwanan ornithomimosaur [30]. Its cervical centra bear small foramina on their lateral surfaces, ventral to the transverse processes (Fig 8). These foramina were noted by de Klerk and colleagues [41] in the initial description, but the authors stated that they could not distinguish if they were pneumatic or vascular. Choiniere and colleagues [30] subsequently confirmed their presence and stated that these foramina were “presumably” (p. 7–8) pneumatic and likely to have also been present on the other cervical vertebrae. Reinspection of the holotype of Nqwebasaurus (AM 6040) has yielded additional morphological information relevant to the evolution of pneumaticity that we summarize here.

The foramen present on the sixth cervical vertebra is small and ovoid, with the long axis oriented anteroposteriorly. It is located between the parapophysis and the transverse process on the lateral side of the anterior end of the centrum, similar to the position and shape of the foramen in *Archaeornithomimus*. Depressions suggesting the presence of similar foramina are also present in cervicals 3–5 but they are filled with matrix, preventing direct confirmation of foramina within the fossae. Without CT data or destructive examination, it is impossible to determine if any of the cervical foramina connected to internal pneumatic chambers. The dorsal surface of the neural arch of cervical vertebra 5 is severely broken, and a mediolaterally narrow, anteroposteriorly long recessed area is exposed and located adjacent to the floor of the neural canal, but separated from this structure by a thin sheet of bone (Fig 8B). This recess is in a topologically identical position to that observed in the CT cross-sections of the cervical vertebrae of *Archaeornithomimus* (Fig 2B), strongly suggesting that *Nqwebasaurus* had pneumatic neural arches.

Four partial dorsal vertebrae are preserved, consisting of two isolated centra, one centrum closely associated with a neural arch, and an isolated neural arch. Of these, only one of the isolated centra was described by Choiniere and colleagues ([30]: Fig 10). All preserved dorsal centra bear a long, ovoid depression on the ventral floor of the neural canal, but this depression does not appear to lead into a pneumatic chamber in the centrum. No external foramina are present on the lateral surface of the dorsal centra. Neither neural arch shows any external foramina or signs of pneumaticity, but the internal structure is unknown. Finally, several broken fragments of neural arch from relatively posterior positions in the axial column are preserved with the holotype materials, and none of these fragments shows evidence of pneumaticity. In summary, the available evidence from *Nqwebasaurus* suggests that it had pneumatic cervical centra and neural arches, apneumatic dorsal centra, and possibly apneumatic dorsal neural arches.

#### Pelecanimimus

The basal ornithomimosaur Pelecanimimus was described by Pérez-Moreno and colleagues ([42]:365) as ‘lacking pleurocoels’ in all presacral vertebrae. However, personal observation of the holotype specimen (LH 7777) reveals that pneumaticity is common in the cervical vertebrae but not clearly present in the dorsal vertebrae (Fig 8C and 8D). The axis has a small ovoid pneumatic foramen (‘pleurocoel’) on the centrum immediately posterior to the parapophysis (Fig 8C) and two potential pneumatic features on the neural arch: a large ovoid fossa immediately above the lamina linking the postzygapophysis and parapophysis, as well as a pocket on the lateral surface of the base of the neural spine (Fig 8D). The third cervical vertebra has a clear pneumatic foramen on the visible side of the centrum but no external signs of pneumaticity on the neural spine (Fig 8C and 8D). Pneumatic features cannot be confidently identified on the more posterior cervicals, which are poorly preserved. However, the lateral surfaces of many of these vertebrae have collapsed posterodorsal to the parapophysis, which suggests that they were excavated in this region, consistent with pneumaticity. Based upon the morphology of cervical 3, pneumatic foramina would be expected in these positions. Pneumatic foramina are absent on all preserved dorsal vertebrae, although a smooth, shallow fossa covers the lateral surfaces of the centra of these vertebrae.

#### Shenzhousaurus

None of the cervical vertebrae are preserved in the only specimen known of the Early Cretaceous ornithomimosaur *Shenzhousaurus* [43]. Only the eight posteriormost dorsal vertebrae are preserved, and none of their centra bear any external evidence of pneumaticity [43]. However, the neural arches of these vertebrae do show external fossae consistent with the possibility of pneumatization, but potential foramina within fossae are obscured by matrix. This was mentioned by Ji and colleagues [43], who found that the prezygapophyseal and postzygapophyseal centrodiapophyseal fossae were pneumatic. Inspection of the holotype (NGMC 97-4-002) shows deep foramina extending anteromedially from the prezygapophyseal centrodiapophyseal fossae into the bases of the prezygapophyses.

**Fig 9:**
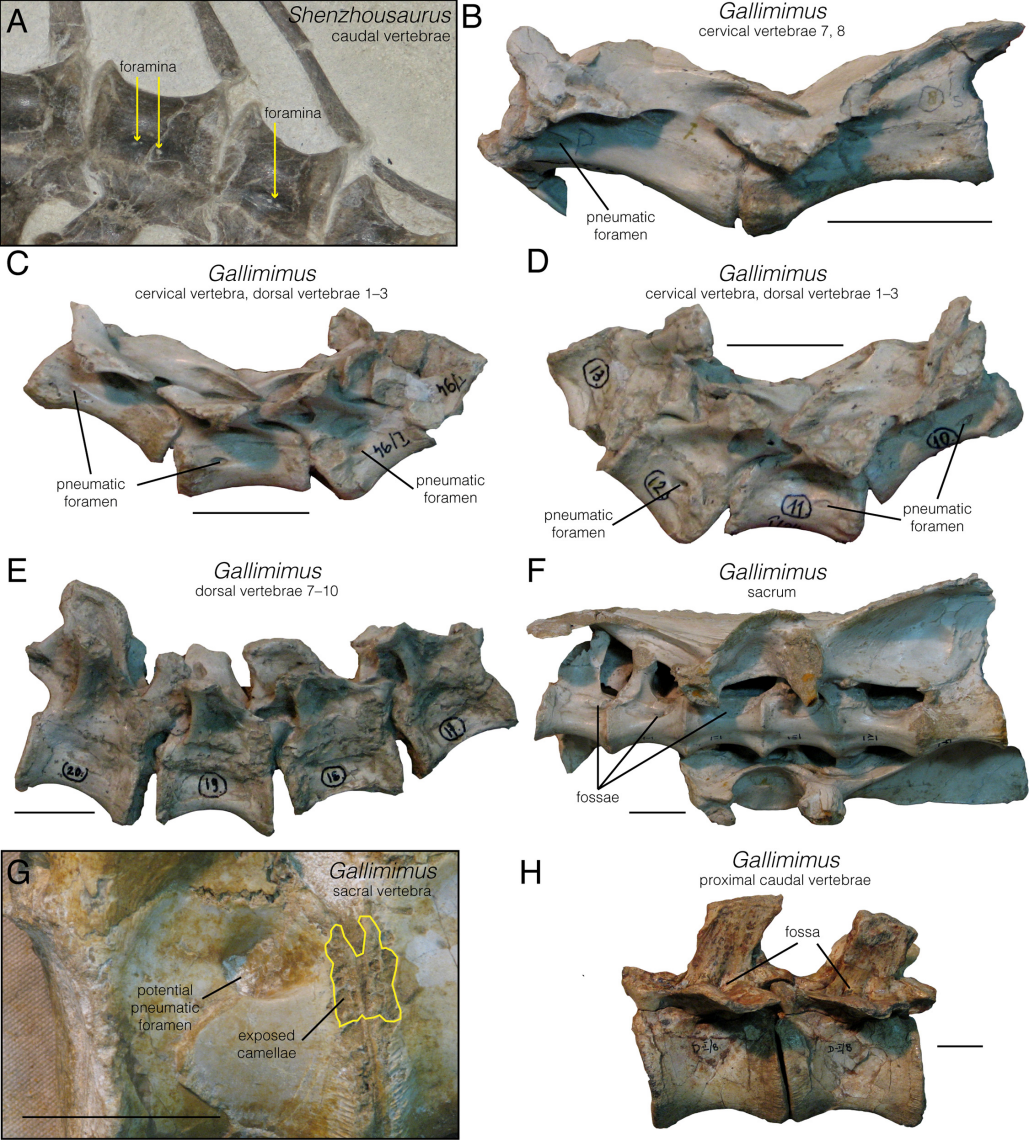
Select vertebrae highlighting the extent of pneumatic structures in *Senzhousaurus* and *Gallimimus*. **A**, proximal caudal vertebrae of *Senzhousaurus* (NGMC-97-4-002), oblique right lateral view; **B**, cervical vertebrae 7, 8 of *Gallimimus* (ZPAL MgD-I/94), left lateral view; **C, D**, left and right lateral views of cervical vertebra and dorsal vertebrae 1, 2 of *Gallimimus* (ZPAL MgD-I/94) respectively; **E**, apneumatic dorsal vertebrae 7–10 of *Gallimimus* (ZPAL MgD-I/94), right lateral view; **F**, sacrum of *Gallimimus* (ZPAL MgD-I/94), left lateral view; **G**, articulated sacral vertebrae of *Gallimimus* (ZPAL MgD-I/29); **H**, proximal caudal vertebrae of *Gallimimus* (ZPAL MgD-I/8), left lateral view. Scale bar equals 3 cm.

The proximal caudal series of *Shenzhousaurus* is well preserved. Deep lateral fossae are present on the proximal seven centra, situated at the level of the neurocentral suture. In the centrum of the third and fourth caudals, the floor of these fossae bears several small, irregularly spaced foramina (Fig 9A). It is unknown whether these connect with internal pneumatic chambers. As such, there is currently no external evidence of pneumaticity in any of the preserved caudal centra.

### Deinocheiridae

#### Garudimimus

**Fig 10:**
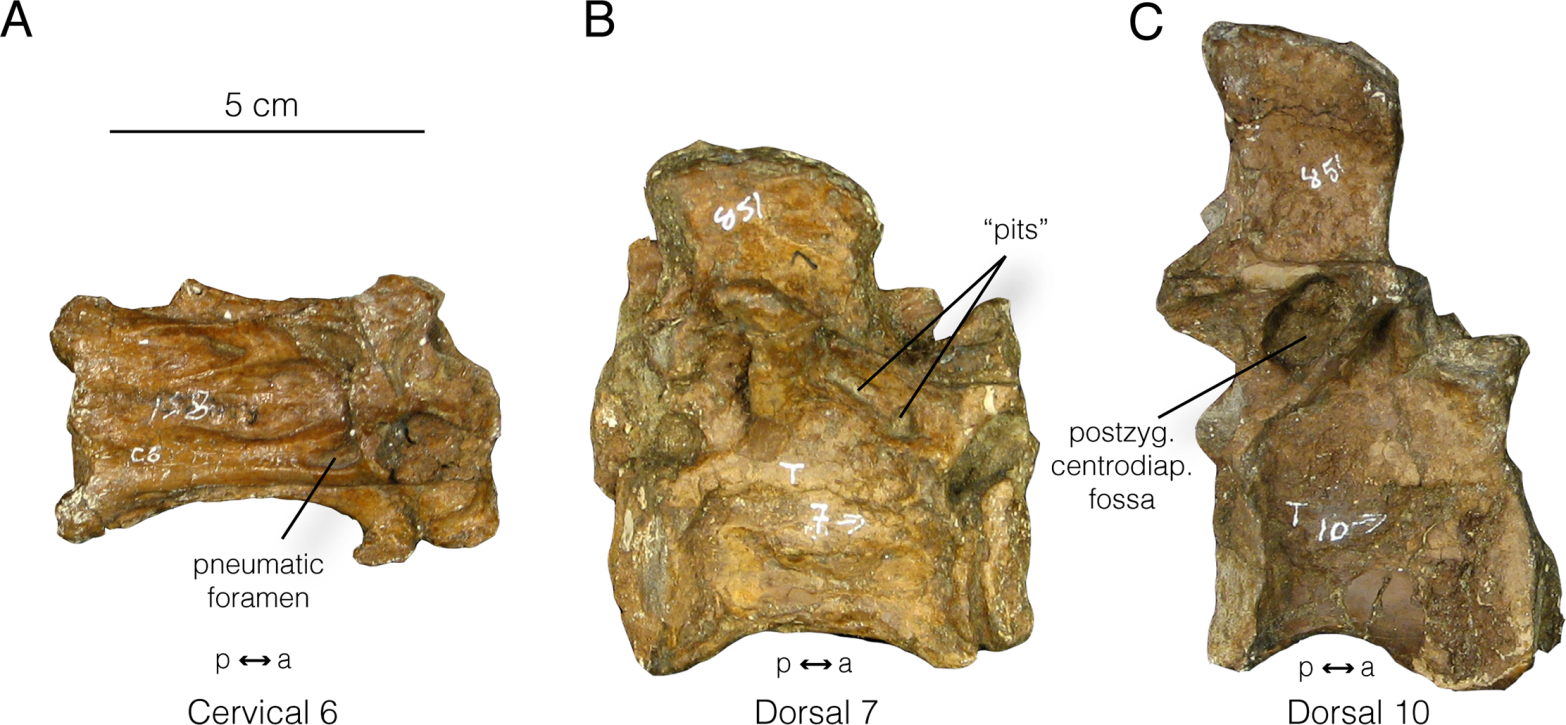
Select vertebrae of *Ornithomimus* (ROM 851). **A**, cervical vertebra 6, right lateral view; **B**, dorsal vertebra 7, right lateral view; **C**, dorsal vertebra 10, right lateral view.

Published information on the presence or absence of pneumatic features in Garudimimus does not allow a definitive assessment. However, Kobayashi and Barsbold ([25]: Fig 10B,G) figured the apparent presence of a large subdiapophyseal foramen on the right side of the neural arch of proximal caudal vertebrae, and of dorsoventrally oriented laminae defining deep fossae on the right dorsolateral surface of a proximal caudal neural arch. These features are similar to those observed in the ornithomimid Gallimimus (Fig 9G) described below and may indicate the presence of caudal pneumaticity in Garudimimus. However, confirmation of this awaits detailed study.

#### Deinocheirus

Lee and colleagues [31] described camellate internal structure in all vertebrae of Deinocheirus, other than the atlas and distal caudals. We regard this account as strong evidence of the presence of pneumaticity in Deinocheirus, which is clearly unlike the condition in other ornithomimosaurs, and might represent the evolution of hyperpneumaticity associated with giant body size [17].

### Ornithomimidae

#### Gallimimus

Osmólska and colleagues [44] described small, deep pleurocoels in the cervical and anterior dorsal centra of Gallimimus, extensive, shallow pleurocoels on middle-posterior dorsal centra, and deep, elongate pleurocoels on the sacral centra. Our observations of multiple specimens (e.g., ZPAL MgD-I/1, MgD-I/94) largely corroborate these observations, although we provide some slight reinterpretations based on the much greater understanding of pneumaticity that has emerged in the four decades since the initial description.

Numerous cervical vertebrae exhibit discrete, ovoid, deeply impressed pneumatic foramina, corresponding to the “small, oval pleurocoels” of Osmólska and colleagues ([44]:118). This is strong evidence of pneumaticity. The best-preserved and most complete cervical series, ZPAL MgD-I/1 and MgD-I/94, show that pneumatic foramina were present on all post-atlantal cervical centra, but no clearly pneumatic features were present on the neural arches (Fig 9B and 9C). Cervical series ZPAL MgD-I/94 is part of a more extensive vertebral series, which also includes a full complement of dorsal vertebrae. The first two dorsal vertebrae exhibit pneumatic foramina on the centra, nearly identical in size and position to those of the cervicals (Fig 9C). These foramina are absent on dorsal 3 and all more posterior dorsals, which instead have broad, shallow fossae on the lateral centrum surfaces (Fig 9C–9E). These are the “extensive, but shallow” pleurocoels of Osmólska and colleagues ([44]:118), but because they do not penetrate the bone and lead into internal chambers, they do not provide definite evidence of pneumaticity [24]. Additional dorsal vertebrae preserved as less complete series corroborate the general observation that pneumatic foramina are present in anterior dorsal centra, but not on more posterior vertebrae (ZPAL MgD-I/1, I/39).

The sacrum of *Gallimimus* is also pneumatic. Osmólska and colleagues [44]:Fig 8 figured one of the best-preserved sacra (ZPAL MgD-I/94). Deep, ovoid, matrix-filled fossae are present on the lateral surfaces of all sacral centra (Fig 9F), corresponding to the “deep, elongate pleurocoels” of [44]. These are distinct from the intervertebral foramina, which occur more dorsally, at the sutures between the neural arches and centra. Furthermore, the deepest portions of these fossae have sharp edges and appear likely to continue into the centrum as foramina (Fig 9). Similar fossae are also seen on other specimens, but vary somewhat in their size and depth. In ZPAL MgD-I/7 large, deeply inset fossae cover nearly the entire lateral surface of each sacral centrum, whereas on ZPAL MgD-I/207 shallow fossae extend across approximately the entire length of the dorsal portion of the centra. The deep penetrating foramina of ZPAL MgD-I/94 are strong evidence of pneumaticity, whereas the deep fossae of ZPAL MgD-I/7 and shallow fossae of ZPAL MgD-I/207 are more equivocal [24]. However, a putative camellate internal structure can be seen on broken abraded surfaces in some specimens, which lends additional evidence of pneumaticity (Fig 9G). Camellate vertebrae have thin external walls, unlike those of camerate and most apneumatic bones [12]. Pneumatic camellae are therefore frequently visible on even weakly abraded bone surfaces in taxa such as carcharodontosaurian, tyrannosauroid, and oviraptorosaurian theropods. They are morphologically distinct from trabecular bone, which cannot be exposed by weak abrasion because it is not typically associated with thin external bone walls. The external exposure of camellae by weak abrasion has thus been regarded as evidence of unambiguous pneumaticity in previous work [17]. It appears that sacral pneumaticity was present in *Gallimimus*, but how this was expressed externally in terms of fossae and foramina is variable between individuals. Further confirmation and characterization of pneumaticity require CT imaging of these sacral vertebrae.

The vast majority of *Gallimimus* caudal vertebrae exhibit no clear signs of pneumaticity (e.g., ZPAL MgD-I/1, I/39, I/94). However, two anterior caudals do possess features that may be pneumatic in origin (ZPAL MgD-I/8): two deep fossae on the web of bone linking the neural spine with the transverse process (Fig 9H). These appear to have foramina inside of them (although this needs to be verified with CT imaging), which if genuine would be strong evidence of pneumaticity. These potentially pneumatic features were not noted by Osmólska and colleagues [44], and may indicate that the proximal portion of the tail was pneumatized in *Gallimimus*. However, this pneumatization would be limited to the neural arch, as pneumatic foramina are not seen on any caudal centra.

#### Ornithomimus

Based on the exemplar specimen ROM 851, well-preserved cervical vertebrae exhibit a deep, ovoid pneumatic foramen at the anteroventral corner of the lateral surface of the centrum, posterior to the parapophysis (Fig 10A). A stout ridge of bone subdivides this foramen internally. None of the dorsal vertebrae, however, show any unequivocal signs of pneumaticity. Pneumatic foramina are absent from all centra. However, on dorsal vertebra 7 there are two pits above the anterior centrodiapophyseal lamina, which potentially could be pneumatic in origin (Fig 10B). In the absence of CT data this is equivocal evidence of pneumaticity, and these pits are not seen on the other dorsal vertebrae. Finally, posterior dorsal vertebra (e.g., dorsal 10) possesses large, deep, funnel-shaped postzygapophyseal centrodiapophyseal fossae (Fig 10C). While this is not by itself unequivocal evidence of pneumaticity, the extreme development of the fossae may be consistent with a pneumatic origin.

## Discussion

### Vertebral pneumaticity in *Archaeornithomimus*

This study provides the first detailed visualization and description of vertebral pneumaticity in a member of Ornithomimosauria, a basally diverging coelurosaurian clade that is crucial for understanding the early evolution of many features traditionally attributed to birds [2,18,28]. Using CT data, we conclude that the neural arches and centra of the postaxial cervical vertebrae, as well as the neural arches of dorsal vertebrae and some proximal caudal vertebrae, are pneumatized (Table 1). Conversely, the centra of dorsal vertebrae, both the neural arch and centra of sacral vertebrae, and the entire mid-distal caudal series lack unambiguous evidence of pneumaticity. The degree of pneumaticity in the atlas and the axis of *Archaeornithomimus* remains unknown because neither is preserved. Notably, our observations include the first evidence of caudal pneumaticity in any ornithomimosaur other than the giant, axially hyperpneumatic *Deinocheirus* [31], contradicting previous generalization that ornithomimosaurs possess apneumatic caudal vertebrae [17]. Although the pattern of internal chambers in the cervical vertebra is largely symmetric, the configuration of pneumatic chambers in the dorsal neural arches is bilaterally asymmetric, including one vertebra in which a chamber is present on one side but absent on the other side. The degree of asymmetry is also different between the two sampled vertebrae, which is congruent with observations of sauropod dinosaur vertebrae [45,46]. This variability could be a manifestation of general plasticity associated with the formation of internal chambers [24,47–54] and mirrors the asymmetry observed among pneumatic features of the crania of theropod dinosaurs [55,56].

**Table 1:** Updated data on the extent of pneumaticity in *Archaeornithomimus*. Bolded cells indicate new or modified information compared to data from [13]. States 0, 1, A, and? indicate absence, presence, ambiguous, and missing respectively. Abbreviations: **C,** centrum; **NA,** neural arch.

Taxon	Atlas NA	Axis NA	Axis C	Post-axial	Anterior dorsal C	Anterior dorsal NA	Mid-posterior dorsal NA	Mid-posterior dorsal C	Sacral NA	Sacral C	Caudal proximal	Caudal middle	Caudal distal
***Nqwebasaurus***	**?**	**?**	**?**	**1**	**0**	**0**	**?**	**?**	**?**	**?**	**?**	**?**	**?**
*Pelecanimimus*	?	**A**	**1**	**1**	**0**	**0**	**0**	**0**	?	?	?	?	?
*Shenzhousaurus*	?	?	?	?	?	?	**1**	0	?	0	**A**	0	?
*Garudimimus*	?	?	0	?	?	?	0	0	?	0	**A**	?	?
***Deinocheirus***	**0**	**1**	**1**	**1**	**1**	**1**	**1**	**1**	**1**	**1**	**1**	**1**	**0**
*Archaeornithomimus*	?	?	?	1	**0**	**1**	0	0	**A**	**0**	**1**	**0**	**0**
*Gallimimus*	?	0	A	1	1	**0**	**0**	**0**	0	**1**	**A**	0	0
*Ornithomimus*	?	?	?	**1**	**0**	**0**	**A**	?	?	?	0	0	0

In addition, the more anterior dorsal vertebra shows clear pneumatic connections between the chambers in the prezygapophyseal and postzygapophyseal centrodiapophyseal fossae that are absent in the more posterior dorsal vertebra in AMNH FARB 21788. These conditions suggest that the more anterior vertebra is more extensively pneumatized than the more posterior dorsal vertebra. Although poor preservation obscures internal structures, CT images of mid- to posterior dorsal vertebrae (AMNH FARB 21788) clearly show equivalent chambers in the neural arch with laminae between the anterior and posterior chambers located in the prezygapophyseal and postzygapophyseal centrodiapophyseal fossae respectively (S2 and S3 Figs). Therefore, the extent of pneumaticity in dorsal vertebrae does not seem to decrease in a regular, graded pattern along the vertebral series. Nevertheless, the termination site of vertebral pneumaticity along the dorsal and sacral vertebrae cannot be determined based on the available specimens.

Both external and internal osteology indicates that sacral vertebrae of *Archaeornithomimus* are apneumatic although the exact length of the apneumatic region (i.e., hiatus) between the dorsal and caudal vertebral series cannot be determined based on the specimens examined here. Two of the more posterior caudal vertebrae (AMNH FARB 21790, 21802) as well as all of the distal caudal vertebrae (AMNH FARB 21794) of *Archaeornithomimus* are also apneumatic. Evolutionary reductions in the extent of pneumaticity have been inferred to be far less common than the independent evolutionary extension of pneumaticity when pneumatic characters are optimized onto a non-avian theropod phylogeny [17]. The pneumatic cervical and anterior dorsal vertebrae of *Archaeornithomimus*, therefore, display a plesiomorphic pneumatic condition requiring no further explanation. However, the internal pneumatic chambers of the caudal neural arches raise questions about patterns of evolution within Ornithomimosauria.

The characterization of vertebral pneumaticity of *Archaeornithomimus* demonstrates that pneumaticity can be subtle and difficult to discern in the absence of CT data, adequate specimen preparation, and the use of microscopy. A potential pneumatic foramen on a sacral vertebra (Fig 4B and 4D), for example, was found to lack a connection to an internal chamber, failing to fulfill the criteria for unambiguous diagnosis of the presence of pneumaticity. In addition, the pneumatic foramina are present on the neural arch rather than the centrum where the presence of a large pneumatic foramen is generally an obvious indicator of pneumaticity. Furthermore, the diameter of pneumatic foramina can be less than a millimeter in diameter, highlighting the importance of careful inspection under microscopy.

### Inferential level of pneumaticity

Our observations on *Archaeornithomimus* allow us to employ and evaluate rules garnered from an extensive survey of pneumaticity in theropod dinosaurs [17]. First, the presence of pneumaticity in any vertebra is always associated with pneumaticity in the cervical vertebrae and anterior dorsal vertebrae (“rule 2” in [17]). For instance, if a caudal vertebra of a taxon is pneumatic, then its cervical and anterior dorsal vertebrae were likely pneumatic as well. Although we did not fully examine the cervical and anterior dorsal axial column, we apply this rule to tentatively conclude that the complete set of cervical vertebrae and anterior dorsal vertebrae were pneumatized in *Archaeornithomimus*. The observed and the inferred presence of pneumatic cervical and anterior dorsal vertebrae strongly suggests the penetration of bones via diverticula from cervical air sacs in *Archaeornithomimus*, following inference based on the strict patterns of regional pneumatization observed in extant birds [16].

Second, pneumatization of the centrum also occurs in sequence from anterior dorsal vertebrae to more posterior vertebrae (“rule 3” in [17]). Again, although we were unable to sample the entire axial column, the application of this rule and our observations that no postcervical centra were pneumatic suggest that the entire set of postcervical centra were apneumatic in *Archaeornithomimus*.

However, Benson and colleagues [17] have also proposed that the posterior extension of pneumaticity from the anterior dorsal vertebrae proceeds in a “neural arch first” pattern where pneumaticity in postcervical vertebrae is always preceded anteriorly by pneumaticity in the neural arch without any gaps (part D of “rule 4”). Thus, if the internal cavities in the neural arch of sacral and caudal vertebrae are in fact pneumatic, then the entire dorsal series were likely pneumatic in the neural arch, but not in the centrum. The observation of pneumatic caudal neural arches in *Archaeornithomimus*, and the absence of pneumatization of the sacrum refutes the proposition that pneumatic hiatuses—the presence of apneumatic vertebrae interrupting series of pneumatic vertebrae—are altogether absent in non-avian theropods (“rule 3” in [17], the ‘no gaps, rule). Wedel and Taylor [46] also demonstrated the presence of pneumatic gaps in the caudal vertebrae of sauropods. Taken together with the known occurrence of pneumatic hiatuses in extant birds, these observations show that “rule 3,” although often true, should not be applied dogmatically in saurischian dinosaurs.

### Evolution of vertebral pneumaticity in ornithomimosaurs

**Fig 11:**
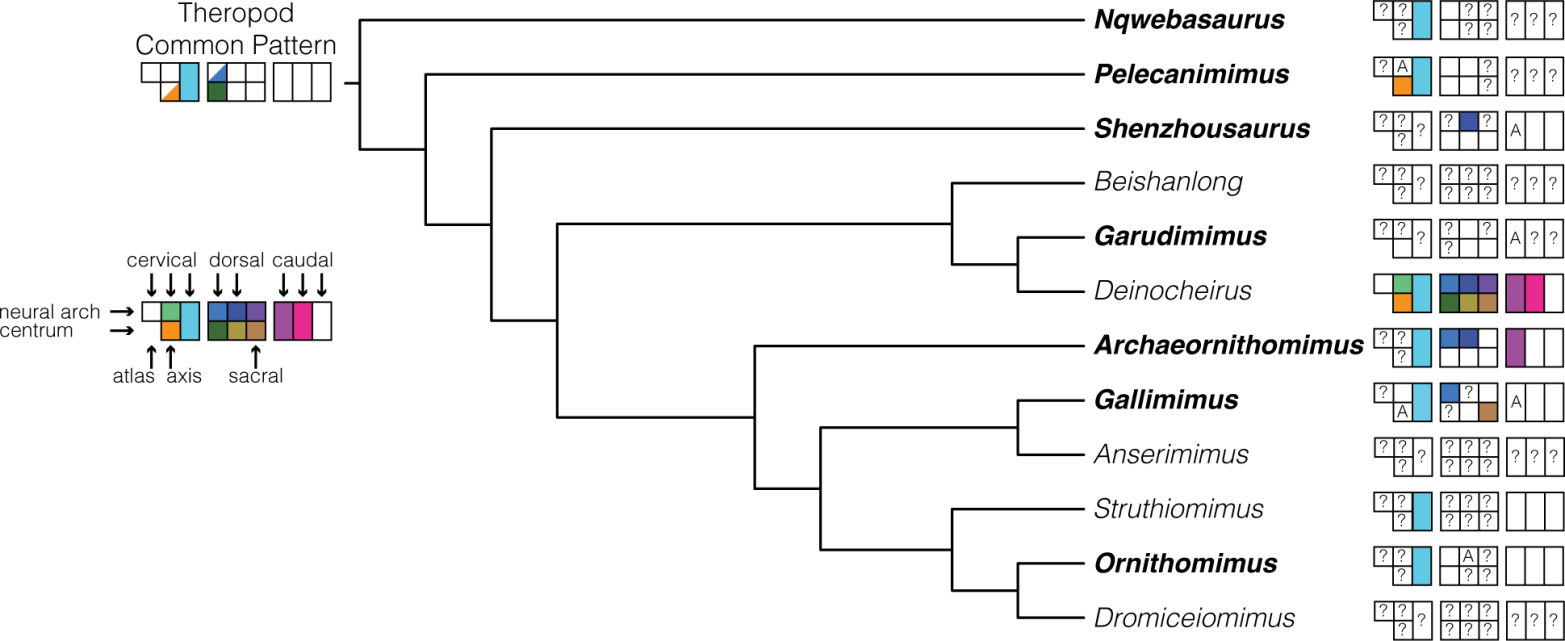
Consensus phylogeny of Ornithomimosauria showing known state of vertebral pneumaticity from the literature and personal observations. Bolded taxonomic names indicate taxa inspected in this study. The common theropod pattern and graphical representation of vertebral pneumaticity is derived from [13]. Colored boxes indicate strong evidence of pneumatization corresponding to the vertebra type and region. Half-filled boxes denote variable pneumatization with respect to the common theropod pattern. “A” signifies ambiguous evidence of pneumatization. “?” indicates no specimen available for the vertebra type and region.

Relative to many other clades of non-avian theropods, ornithomimosaurs collectively exhibit a lower degree of postcranial pneumaticity (Fig 11). Extensive pneumaticity in the caudal vertebrae, for instance, evolved independently in multiple non-avian theropod clades, including megalosaurids, allosauroids, therizinosauroids, and oviraptorosaurs ([17] and references therein). Pneumatized sacral vertebrae also occur in some, but not all, abelisauroids, allosauroids, tyrannosauroids, therizinosauroids, oviraptorosaurs, and troodontids [16,17]. These taxa show an “extended pattern” of vertebral pneumaticity from the phylogenetically optimized primitive theropod and avian conditions, in which pneumaticity is limited to postaxial cervical and anterior dorsal vertebrae [17,53,57].

Unfortunately, limited knowledge of the distribution of postcranial pneumaticity in ornithomimosaurs still hinders interpretation of its evolution within the group (Table 1; Fig 11). We can, however, infer some macroevolutionary trends in vertebral pneumaticity of ornithomimosaurs. First, the basal members *Nqwebasaurus* and *Pelecanimimus* exhibit the common ancestral pattern of pneumaticity in cervical and often also anterior dorsal vertebrae that occurs across basal members of major theropod groups as reported by Benson and colleagues [17]. This observation suggests that, as in many other theropod lineages, ancestral members of Ornithomimosauria possessed a plesiomorphic condition with pneumatic cervical vertebrae, and perhaps even less pneumaticity due to apparently apneumatic dorsal vertebrae in *Nqwebasaurus* and *Pelecanimimus*.

Second, *Archaeornithomimus* shows far greater vertebral pneumaticity than other ornithomimosaurs with the exceptions of *Gallimimus* and *Deinocheirus*, which has pneumatic sacral vertebrae, mid-caudal vertebrae, and centra of dorsal vertebrae [31]. Limited vertebral pneumaticity in the basal deinocheirid *Garudimimus* implies that ornithomimids (*Archaeornithomimus*, *Gallimimus*) and *Deinocheirus* acquired elevated degrees of pneumaticity independently. Despite possessing greater postcranial pneumaticity than basal ornithomimosaurs, such as *Pelecanimimus* and *Nqwebasaurus*, *Archaeornithomimus* shows limited vertebral pneumaticity relative to other theropod taxa possessing pneumatic caudal vertebrae, which typically have pneumatic dorsal and sacral neural arches, and often centra [17].

Based on current limited data, other ornithomimids, including *Ornithomimus* and *Struthiomimus*, lack evidence of pneumaticity in caudal vertebrae and potentially anterior dorsal vertebrae in *Ornithomimus*. If *Archaeornithomimus* is a basally diverging member of the family Ornithomimidae, then the ornithomimids may have ancestrally possessed pneumatic neural arches in the dorsal vertebrae, which were subsequently lost in more derived ornithomimids. This inference, however, relies on the absence of evidence for pneumaticity in the dorsal neural arches of poorly documented fossils, rather than on proper evidence of absence. Currently, we cannot conclusively characterize the level of vertebral pneumaticity in these taxa, and thus, the conditions seen in *Archaeornithomimus* and *Gallimumus* may be more widespread within Ornithomimidae. Clearly, additional data on vertebral pneumaticity, particularly through CT imaging, are needed to generate more precise and conclusive statements about the evolutionary history of axial pneumaticity in Ornithomimosauria, as well as across bird-line dinosaurs.

### Evolution of abdominal air sacs

The presence of a hiatus in vertebral pneumaticity has relevance to the evolution of air sacs that characterize modern birds. Extant birds possess up to nine air sacs: a single clavicular air sac and paired cervical, anterior thoracic, posterior thoracic, and abdominal sacs [58–60]. Of these, the cervical and abdominal air sacs invade the axial skeleton. Cervical air sac diverticula invade cervical vertebrae and the anterior-most dorsal vertebrae, as well as ribs, and do not contribute to respiratory function [59–61]. In extant birds, abdominal air sacs located posteriorly along the body profile generally pneumatize the synsacral vertebrae.

Although cervical air sac seems to have evolved early in the theropod lineage, the origin of the abdominal air sacs is a contentious issue in archosaur paleontology (e.g., [12,19,24,53]). Some researchers have advocated the presence of abdominal sacs in *Archaeopteryx* due to the presence of a pneumatic pelvis [62] and in the abelisauroid *Majungasaurus* [16]. These inferences have been supported by systematic study of 234 extant bird species, including the turkey, *Meleagris*, which demonstrated that “in no cases do cervical air-sac diverticula extend caudally along the column to pneumatize vertebrae beyond the middle thoracic series” ([16], p. 253), although it had previously been reported that the synsacral vertebrae in turkeys were invaded by cervical air sacs [63]. Others have argued that pneumaticity in the posterior axial skeletons of non-avian theropods could have been implemented by posterior extensions of the cervical air sacs (e.g., [22,27]) citing the fact that none of the non-avian theropods possess a hiatus in pneumatic foramina along the vertebral series. In crown group birds, the presence of such a hiatus is a sign of the division between cervical and abdominal air sacs.

Pneumatic hiatuses, therefore, have played an influential role in discussions of the inference of an avian-like respiratory anatomy in non-avian theropods [16,19,20,24,64]. Specifically, they have been thought to provide evidence of multiple sources of pneumatization located at different points along the axial column, and therefore the presence of multiple air sacs in a potentially avian-like configuration. Small hiatuses are present in the axial columns of some sauropods [46,64]. However, until now, the only tentative evidence of this occurring in non-avian theropods has come from differences in the sizes of pneumatic foramina along the axial column of the ceratosaur *Majungasaurus*, which lacks any actual pneumatic hiatuses [16,17].

The presence of an apparent pneumatic hiatus between the dorsal and caudal vertebrae of *Archaeornithomimus*, revealed by CT scanning, suggests that evidence of hiatuses in other taxa, and further key observations on the distribution of postcranial pneumatization in the avian stem lineage, might have been overlooked by previous workers. The pneumatic hiatus of *Archaeornithomimus* points to the possibility that abdominal air sacs, distinct from cervical air sacs, were present in ornithomimosaurs, and may characterize the evolution of the earliest coelurosaurs. Alternatively, abdominal air sacs could have appeared earlier during theropod [16], saurischian (e.g., [19,64]), or ornithodiran [14] evolution, but only rarely have been manifested in the form of distinct pneumatic hiatuses. Although the present specimen is the only other known instance of a hiatus in non-avian theropods, such gaps may become more widely recognized as CT data are more widely employed in the study of vertebral pneumatization.

## Conclusions

Using μCT imaging, this study provides the first comprehensive report on the vertebral pneumaticity in not only a member of Ornithomimosauria, but a non-avian coelurosaur. Both internal and external osteology demonstrates the conspicuous and definitive presence of pneumaticity in cervical and anterior dorsal vertebrae. In contrast, the sacral vertebrae completely lack clear signs of pneumaticity. Notably, *Archaeornithomimus* exhibits one of the first documented cases of pneumaticity in the caudal vertebrae among ornithomimosaurs alongside *Deinocheirus*, although these features are subtle and restricted to the neural arch. Although speculative, the presence of a pneumatic hiatus in the sacral vertebrae between the dorsal and caudal vertebrae may suggest that a separate abdominal air sac supplied the caudal vertebrae.

Aside from the partly pneumatic caudal vertebrae, *Archaeornithomimus* conforms to a plesiomorphic postcranial pneumatic condition. When compared to other major theropod clades, ornithomimosaurs exhibit limited vertebral pneumaticity with the exception of *Deinocheirus*. A survey within Ornithomimosauria suggests that vertebral pneumaticity was restricted to cervical vertebrae in ancestral ornithomimosaurs and subsequently extended to postcervical vertebrae in Deinocheiridae and Ornithomimidae independently. Possible evidence of a reduction in vertebral pneumaticity among more derived ornithomimids supports the notion that the extent of postcranial pneumaticity is homoplasious within ornithomimosaurs and, as shown previously [17], among theropods.

With CT data, these vertebrae reveal a degree of individual variation, including asymmetry in both external and internal pneumatic structures. This observation complements previous studies on extant birds documenting high individual and ontogenetic variations in pneumaticity [46,52,65]. If possible, multiple individuals should be examined to accurately characterize the degree of both intra- and interspecific variation in pneumaticity. Unfortunately, this is often difficult to achieve with fossil materials. Nevertheless, we encourage the use of CT imaging on fossil vertebrae to characterize their pneumaticity with greater fidelity and precision, an endeavor necessary to further elucidate the evolutionary transformation of postcranial pneumaticity in bird-line archosaurs.

## Supporting Information

S1 Fig Two small probable neurovascular foramina (arrows) in the left centrodiapophyseal fossa of the more anterior vertebra in AMNH FARB 21788. (TIF)

S2 Fig CT images of posterior dorsal vertebra of *Archaeornithomimus* (AMNH FARB 21788). A, transverse section; B, midsagittal section; C, frontal section.
(TIF)

S3 Fig CT images of posterior dorsal vertebra of *Archaeornithomimus* (AMNH FARB 21788). A, transverse section; B, midsagittal section; C, frontal section.
(TIF)

S1 File CT video of cervical vertebra of *Archaeornithomimus* (AMNH FARB 21786) from anterior to posterior (Video A), from dorsal to ventral (Video B), and from left to right (Video C). (ZIP)

S2 File CT video of dorsal vertebrae of *Archaeornithomimus* (AMNH FARB 21788) from anterior to posterior (Video A), from dorsal to ventral (Video B), and from left to right (Video C). (ZIP)

S3 File CT video of sacral vertebrae of *Archaeornithomimus* (AMNH FARB 21790) from anterior to posterior (Video A), from dorsal to ventral (Video B), and from left to right (Video C). (ZIP)

S4 File CT video of caudal vertebrae of *Archaeornithomimus* (AMNH FARB 21790) from anterior to posterior (Video A), and from anterior to posterior (Video B). (ZIP)

S1 Table Specimens used in this study and corresponding settings for CT imaging. (DOCX)
