# Supplementary figures and images for: Vertebral Pneumaticity in the Ornithomimosaur Archaeornithomimus (Dinosauria: Theropoda) Revealed by Computed Tomography Imaging and Reappraisal of Axial Pneumaticity in Ornithomimosauria
Source: PLoS One. 2015 Dec 18;10(12):e0145168. doi: 10.1371/journal.pone.0145168 (PMC4684312; doi:10.1371/journal.pone.0145168)

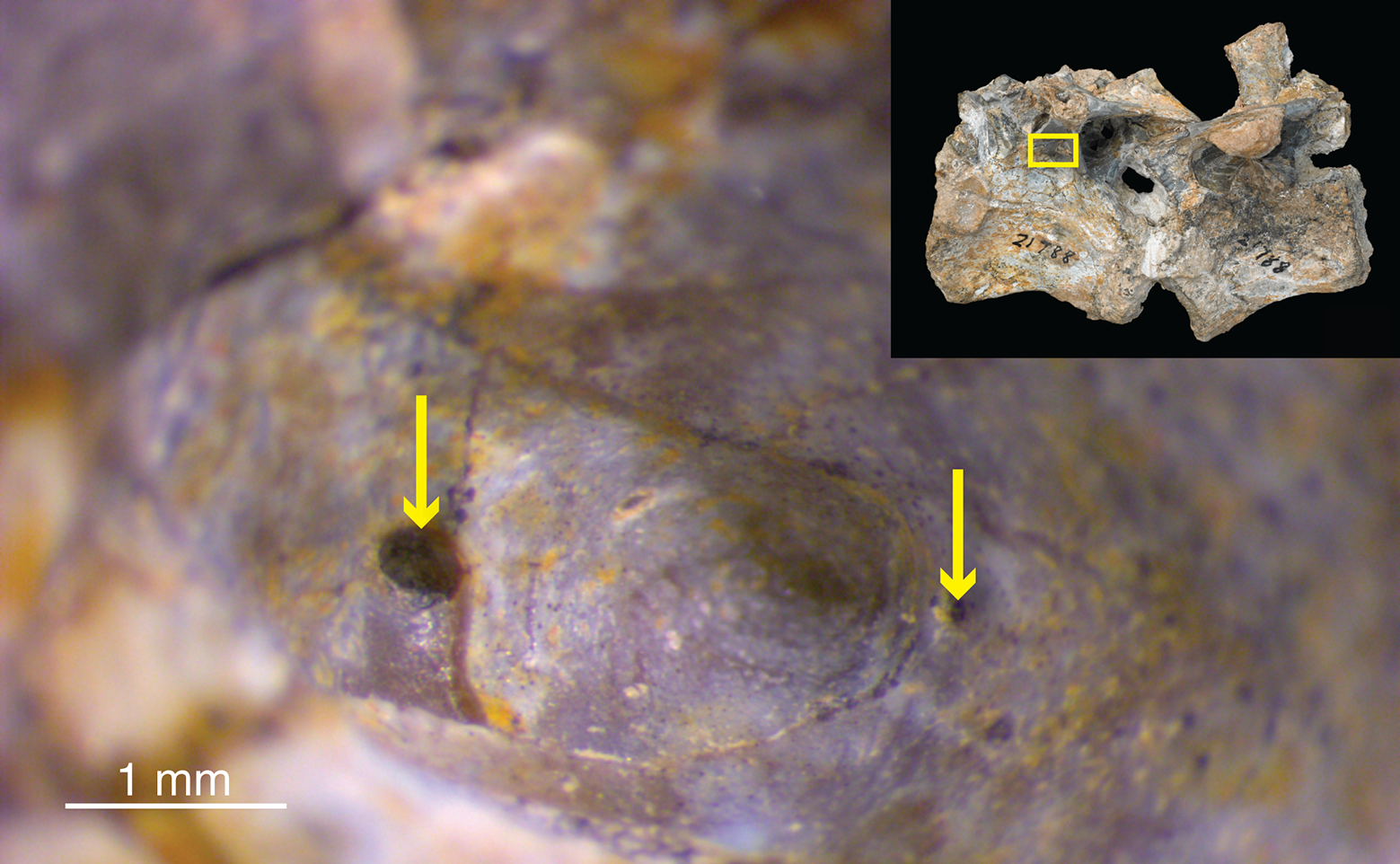

Supplement: S1 Fig — (TIF) [file pone.0145168.s001.tif]

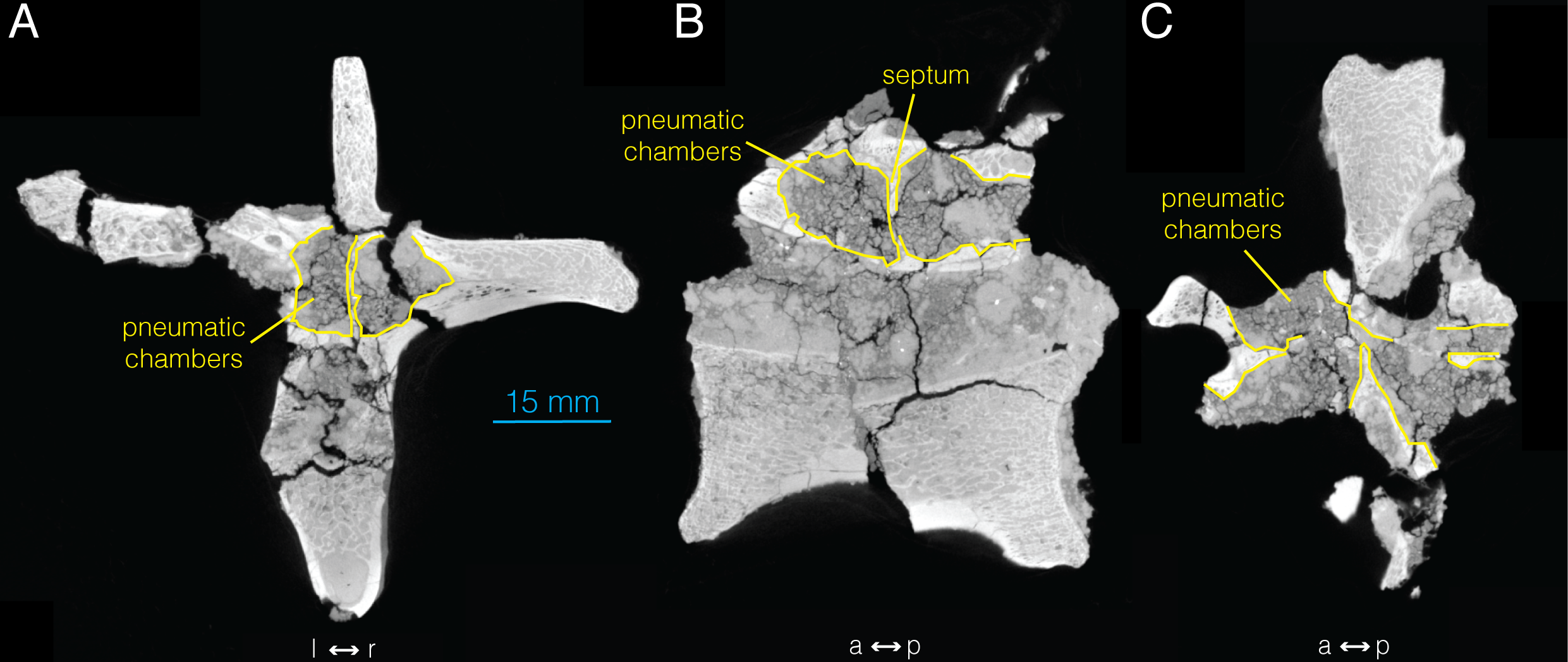

Supplement: S2 Fig — A, transverse section; B, midsagittal section; C, frontal section. (TIF) [file pone.0145168.s002.tif]

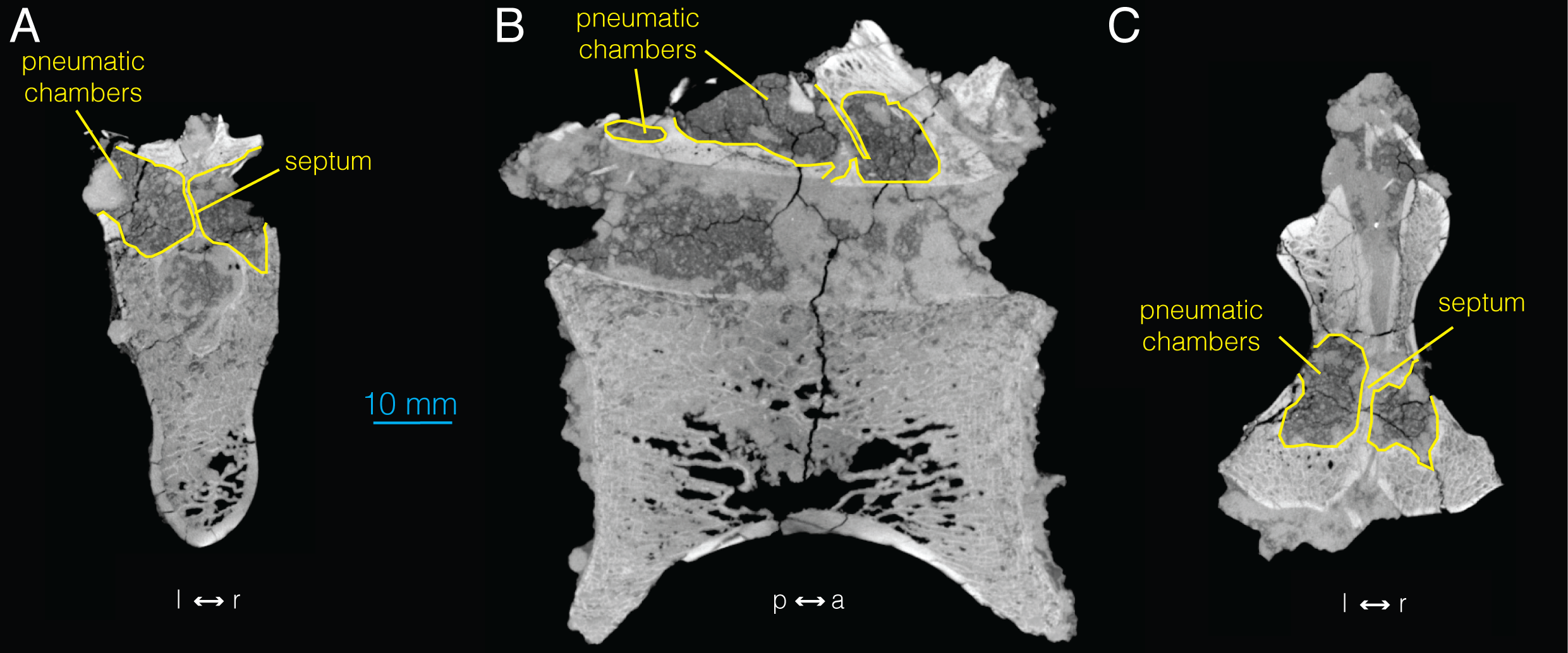

Supplement: S3 Fig — A, transverse section; B, midsagittal section; C, frontal section. (TIF) [file pone.0145168.s003.tif]
