# Supplementary material for: Vertebral Pneumaticity in the Ornithomimosaur Archaeornithomimus (Dinosauria: Theropoda) Revealed by Computed Tomography Imaging and Reappraisal of Axial Pneumaticity in Ornithomimosauria
Source: PLoS One. 2015 Dec 18;10(12):e0145168. doi: 10.1371/journal.pone.0145168 (PMC4684312; doi:10.1371/journal.pone.0145168)
Supplement: S1 Table — (DOCX) [file pone.0145168.s008.docx]

TABLE S1: Specimens used in this study and corresponding settings for computed tomography (CT) imaging.

| Vertebra type | Specimen no. | Voltage (kV) | Current (μA) | Voxel size (μm) |
| --- | --- | --- | --- | --- |
| Cervical | AMNH FARB 21786 | 170 | 150 | 101.2571 |
| Dorsals | AMNH FARB 21788 | 170 | 150 | 117.5757 |
| Sacrals | AMNH FARB 21790 | 170 | 150 | 120.9089 |
| Proximal caudals | AMNH FARB 21790, 21802 | 170 | 150 | 84.86221 |
| Distal caudals | AMNH FARB 21794 | 220 | 260 | 90.34096 |
